# GWAS identifies a polyembryony locus in mango: development of KASP and PACE markers for marker-assisted breeding

**DOI:** 10.3389/fpls.2025.1508027

**Published:** 2025-01-29

**Authors:** Gul Shad Ali, Shamseldeen Eltaher, Jin Li, Barbie Freeman, Sukhwinder Singh

**Affiliations:** ^1^ Subtropical Horticulture Research Station (SHRS), United States Department of Agriculture, Agricultural Research Service (USDA-ARS), Miami, FL, United States; ^2^ Department of Plant Biotechnology, Genetic Engineering and Biotechnology Research Institute (GEBRI), University of Sadat City (USC), Sadat City, Egypt

**Keywords:** GWAS, MAS, KASP, PACE, mango, polyembryonic mango, apomixis

## Abstract

Apomixis is a horticultural trait that enables clonal propagation of hybrids by producing asexual embryos from maternal cells in the ovule without meiosis. Many mango cultivars exhibit apomictic polyembryony, where one embryo develops from zygotic tissues and the rest from nucellar tissues, resulting in seedlings that are genetically identical to the mother tree. In *Mangifera indica* L. commercially important rootstocks are raised from apomictic seeds, which are then grafted with desired cultivars. Identifying molecular markers for polyembryony and understanding its genetics would facilitate introducing this trait in commercially important cultivars. In this report, genome-wide association studies were conducted on a diversity panel consisting of 42 polyembryonic and 42 monoembryonic *M. indica* cultivars using high-density single nucleotide polymorphism (SNP) markers. These studies revealed that the polyembryony locus is in a 360-kb region on linkage group 17 of the ‘Alphonso’ reference genome. This locus contains the *MiRWP/MiRKD4* gene, which codes for an RWP–RK domain-containing protein previously implicated in citrus apomixis. Comparative genomic analyses revealed synteny between the citrus and the mango polyembryony loci, suggesting a common evolutionary mechanism for this trait. A total of 29 SNP markers in this locus were significantly associated with polyembryony in *M. indica*. Five of these markers were developed into convenient genotyping assays using competitive allele-specific PCR chemistry implemented in two different genotyping platforms – Kompetitive Allele-Specific PCR (KASP) and PCR allele competitive extension (PACE). The utility of these assays was validated and demonstrated in diverse germplasm collection and open-pollinated mango breeding populations with known pedigrees and polyembryony phenotypes. These SNP markers, especially those flanking the *MiRWP/MiRKD4* gene, provide a valuable tool for mango breeders to select polyembryonic progenies at the seedling stages in mango breeding programs.

## Introduction

Mango (*Mangifera indica* L.) is a significant fruit crop known for its large size and soft, sweet pulp. Originally native to South and Southeast Asia, Mangos are now popular in tropical and subtropical regions all over the world. In 2021, the output volume of mango, mangosteen, and guava increased slightly from over 56.69 million metric tons in 2020 to just over 57 million metric tons (https://www.statista.com/statistics/577951/world-mango-production/). Southeast Asia, including India and several other countries, is the primary mango-producing region. Over the past few centuries, hundreds of cultivars have been selected in various mango-growing regions, primarily in India and the Pacific islands (Mukherjee and Litz, 2009). During the late 19th and early 20th centuries, new mango varieties were selected as chance seedlings in Florida. By breeding for red blush coloration, mild taste, and mild aroma ideotype, these cultivars are adapted to consumers’ tastes from different demographics. Still, there is a need for cultivar improvement, and breeding programs are currently underway in Australia, South Africa, Brazil, and Israel (Olano et al., 2005; Jain et al., 2009; Sherman et al., 2015). Mango cultivars can be classified as either monoembryonic or polyembryonic depending on the type of embryo and the number of embryos contained in each seed (Iyer and Subramanyam, 1989; Mukherjee and Litz, 2009; Song et al., 2023). Monoembryonic cultivars are suggested to have originated in the Indian subcontinent, while polyembryonic cultivars in Southeast Asia (Mukherjee and Litz, 2009).

Based on numerous studies (Duval et al., 2005; Schnell et al., 2005; Viruel et al., 2005), it is now well established that genetic markers in Mangos are inherited in a disomic fashion, and as a result, Mangos should be considered diploid. Based on cytogenetics, the mango genome has been suggested to be a partially allopolyploid (Mukherjee, 1950). Based on genetic linkage analyses, mango has 40 chromosomes (2n=40, x=20) and 20 linkage groups (Duval et al., 2005; Schnell et al., 2005; Wang et al., 2020; Ma et al., 2021), demonstrating that Mangos are diploid. The estimated size of the haploid genome is approximately 439 Mb (Arumuganathan and Earle, 1991; Kuhn et al., 2017), with a reference genome size of 392 Mb (Wang et al., 2020).

Polyembryony is a form of apomixis in which multiple somatic embryos develop without meiosis from nucellar cells (Koltunow, 1993; León-Martínez and Vielle-Calzada, 2019). Apomixis has been reported in at least 78 families and 293 flowering plant genera (https://uni-goettingen.de/en/423360.html). Polyembryony is considered a desirable trait as it allows clonal propagation of desirable hybrids, especially uniform rootstocks, which would otherwise differ phenotypically from the mother plant if produced zygotically. However, because nucellar seedlings only reveal maternal genotypes, it is necessary to separate zygotic embryos from apomictic embryos to draw valid findings from genetic analyses of segregating progenies.

Polyembryony is a desirable trait in *Mangifera* spp. propagation for maintaining the genetic identity of the rootstock parent. *M. indica* cultivars are highly heterozygous and are vegetatively propagated by grafting on a suitable rootstock. Initially, polyembryony in mango was reported to be controlled by two recessive loci (Sturrock, 1968). However, this finding was refuted through carefully designed genetic analyses, which showed that a single dominant locus controls polyembryony in Mangos (Aron et al., 1998; Kuhn et al., 2017). Similarly, a single dominantly inherited polyembryony locus controls polyembryony in various citrus and related species, further supporting the single dominant polyembryony locus (Iwamasa, 1967; Nakano et al., 2012). Extensive genomic analyses of polyembryonic and monoembryonic citrus accessions, including wild, primitive, and cultivated species, as well as genetic studies of a segregating population of citrus, have shown that polyembryony is caused by the insertion of Miniature Inverted-repeat Transposable Elements (MITE) in the promoter of *CitRWP* gene (Wang et al., 2017). Another study independently corroborated these results, which also reported that insertion of MITE in the promoter of the same gene, referred to as *CitRKD*, controlled polyembryony in citrus (Shimada et al., 2018). Furthermore, Shimada et al (Shimada et al., 2018), also suggested that the insertion of MITE in one of two alleles of *CitRKD1* lead to predominant expression in reproductive tissues. Further support for the involvement of *CitRWP/CitRKD1* in polyembrony was provided in the same study by demonstrating that the genetic transformation to knock out the function of *CitRKD1* resulted in the loss of polyembryony. In a recent study, sequencing and phylogenetic characterization of the MITE in citrus suggested that the polyembryony locus is complex, consisting of varying numbers of MITEs, and that it might have evolved from a common ancestor of citrus (Shimada et al., 2018). CitRWP/CitRKD1 belongs to the plant RWP-RK domain-containing protein family and is a homolog of the *Arabidopsis* RKD family gene. It is mostly transcribed in the reproductive organs of polyembryonic types (Shimada et al., 2018), suggesting that it might be controlling the expression of key genes in embryogenesis. During the preparation of this manuscript, Yadav et al (Yadav et al., 2023), reported that polyembryony in mango is also controlled similarly by this gene through SNP genotyping analysis of 200 accessions from germplasm collections in Israel and Australia and whole genome sequencing of 15 accessions. They employed a comparative genomics approach and suggested a homologous gene to *CitRWP* was implicated in mango’s polyembryony. Our study corroborated these findings. Leveraging statistically robust genome-wide association studies, we analyzed high-coverage whole-genome resequencing data from 42 monoembryonic and 42 polyembryonic varieties sourced from the USDA ARS Subtropical Horticultural Research Station germplasm collection in Miami, Florida, USA. Our findings revealed a greater density of single nucleotide polymorphisms (SNPs) associated with polyembryony. Additionally, we found that some cultivars are homozygous, whereas others are heterozygous for several of the significant SNPs.

In our studies, we conducted high-quality whole-genome resequencing to attain an average genome coverage of over 25-fold, which resulted in identifying high-quality 133,590 SNP. Using these high-density SNP markers on a panel of 84 genetically diverse *M. indica* accessions, which were categorized into polyembryonic and monoembryonic sets, we aimed to precisely map the polyembryonic locus using genome-wide association studies (GWAS). The SNP data were also used to analyze the population structure and evolutionary relationships of the 84 mango accessions collected globally, and an in-depth comparison of the polyembryonic regions in these accessions was conducted. Comparative genomic analysis methods demonstrated synteny and strong collinearity around the polyembryonic locus in mango and citrus. This indicates a conserved evolutionary genetic basis for polyembryony in *Mangifera* spp and citrus, emphasizing the potential implications of these findings in understanding and harnessing this trait for crop improvement. For practical application in breeding programs, five of these markers, located upstream and downstream of the *MiRWP/MiRKD4* gene were developed into two competitive allele-specific PCR assays. The utility of these markers was validated in breeding populations resulting from hybridization of monoembryonic and polyembryonic parents.

## Results

### Geographical distribution, whole genome sequencing and single nucleotide polymorphism analyses of *M. indica* cultivars

**Figure 1:**
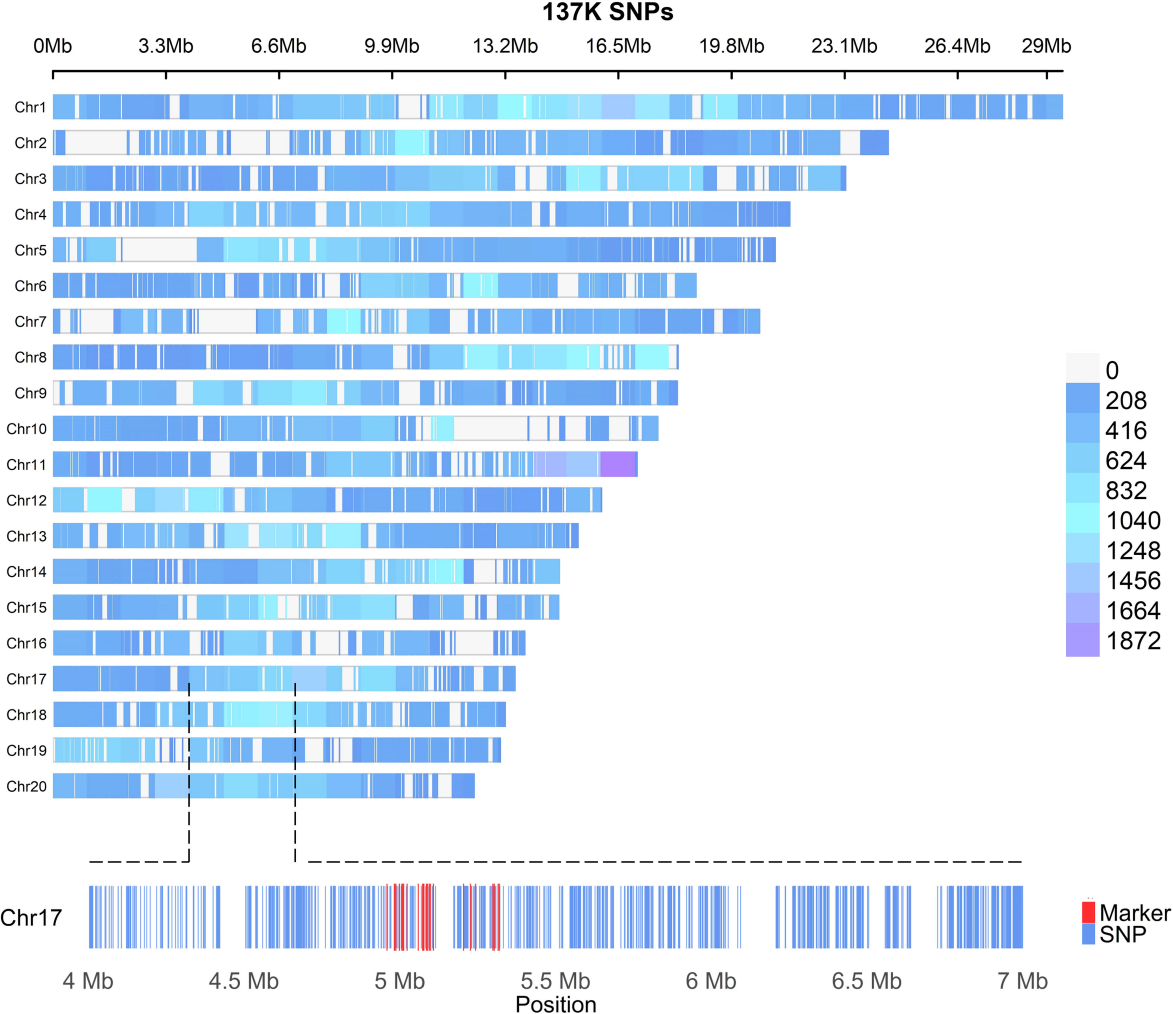
Density plot of the 133,590 SNPs mapped to the 20 chromosomes of the *M. indica* Alphonso reference genome CATAS_Mindica_2.1 (GCA_011075055.1). Each hyphen represents one SNP. The distribution density is shown by different colors, with blue indicating low and purple indicating high. In the zoomed-in view of chromosome 17, each blue hyphen corresponds to a SNP. Markers significantly associated with polyembryony in *M. indica* are highlighted in red.

The USDA-ARS, Subtropical Horticulture Research Station (SHRS), Miami, FL maintains 278 *M. indica* accessions from different parts of the world (**Supplementary Figure S1A**). To determine the embryony status, mature fruits (*n* = 12-36) were collected from different *M. indica* cultivars and examined for the number of embryos. Seeds with two or more embryos were considered polyembryonic. Polyembryony in *Mangifera* species is usually associated with Southeast Asian mango cultivars, whereas monoembryony is more common in Indian cultivars (Shukla et al., 2004). Among the polyembryonic accessions, frequency of polyembryonic fruit ranged from 33-100% (**Supplementary Figures S1B, C**) suggesting that polyembryony in mangos is facultative with varying penetrance levels, which might be dependent on genotype x environment interactions similar to what has been reported for the level of polyembryony in citrus (Khan and Roose, 1988). For whole genome resequencing, 42 polyembryonic and 42 monoembryonic accessions were selected from the mango clonal collection at SHRS and sequenced using the Illumina 150 bp paired end reads to an average depth of 25X genome coverage based on the 392 Mb genome size of *M. indica* cultivar ‘Alphonso’. A total of 134,251 high-quality biallelic SNPs (minor allele frequency > 0.05 and missing data < 0.1) were selected and used for population genetic analyses and marker identification for polyembryony. Of these SNPs, 133,590 were successfully mapped to the 20 chromosomes of the ‘Alphonso’ reference genome, as illustrated in **Figure 1**. Marker density varied in different chromosomes with an average high density of 1 SNP/2.678kb. Most of these SNPs were found in the intergenic and intronic regions (**Supplementary Figure S2**). The nonsynonymous/synonymous ratio (ω = dN/dS) was 1.05, indicating a slight overrepresentation of nonsynonymous substitutions. This observation suggests a weak positive selection, signifying potential adaptive evolution in the associated genomic regions.

### Population structure and linkage disequilibrium analyses

**Figure 2:**
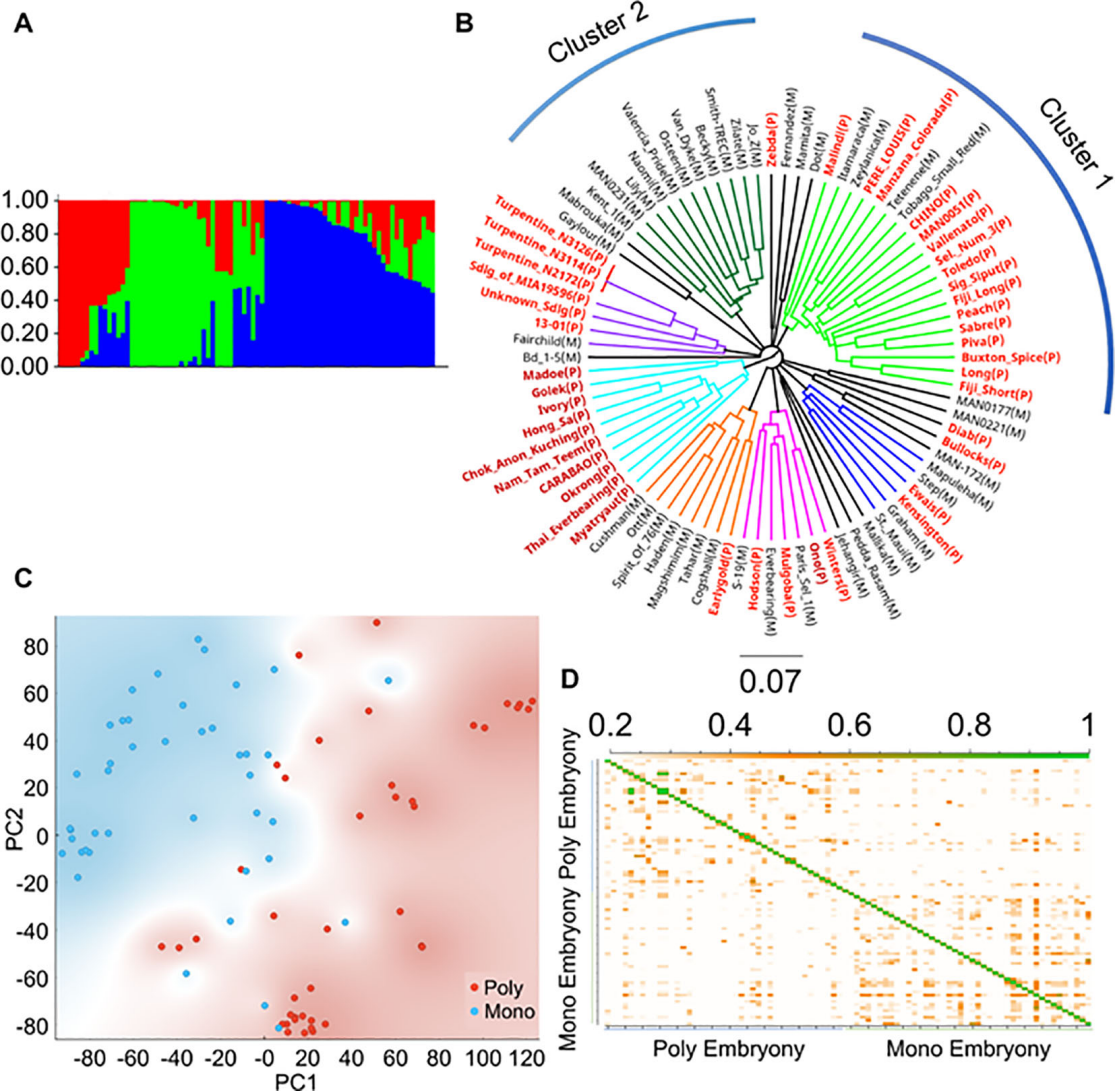
Population analysis with SNP data of 84 *Mangifera indica* accessions. **(A)** Genetic population structure analysis exhibits three distinct groups among the 84 mango accessions. **(B)** Phylogenetic tree of mango accessions based on high-quality SNPs reveals several distinct clusters, in which polyembryony represented by red color font of the cultivar names with (P) is common in Southeast Asian accessions and monoembryony represented by black font with (M) is prevalent in Indian and American cultivars. **(C)** Principal component analysis shows a similar structure to the phylogenetic tree, with monoembryonic and polyembryonic varieties grouped roughly along diagonals. Blue color represents monoembryonic and red color represent polyembryonic cultivars. **(D)** Identity by descent (IBD) plot of monoembryonic and polyembryonic cultivars. IBD values ranged from 0.2 - 0.5 and are shown in orange, indicating close kinship. Identical clones are shown in green color.

Population structuring analyses using STRUCTURE, phylogenetic analyses, and principal component analyses (PCA) revealed several clusters in the cultivars used in this study. Structure analyses divided the populations into roughly three groups (**Figure 2A**, *k* = 3; **Supplementary Figure S3**). The Southeast Asian accessions generally clustered together, as shown in red (**Figure 2A**). Phylogenetic analyses revealed seven major well-supported clusters (**Figure 2B**). Consistent with their Southeast Asian origin, most cultivars in cluster 1 displayed polyembryony (**Figure 2B**). In contrast, cultivars of U.S. origin, which mostly grouped in cluster 2, displayed monoembryony. The remaining clusters, consisting of cultivars of an admixed nature, had both poly and monoembryony. PCA showed similar results, with the polyembryonic accessions shown in red grouping on one side of the PCA plot and monoembryonic in blue on the other (**Figure 2C**). Identity-by-descent (IBD) analyses revealed that most accessions displayed less relatedness (shown as white background, IBD estimates < 0.2) (**Figure 2D**). Levels of interrelatedness among accessions within the monoembryonic and polyembryonic groups were higher than among the polyembryonic and monoembryonic groups (shown as orange squares, IBD estimates: 0.2 - 0.5). According to the linkage disequilibrium (LD) decay plot (**Supplementary Figure S4**), the genome-wide average *r^2^* value was 0.181 for the monoembryonic group, which occurred at a physical distance of 104 kb, and it was 0.163 for the polyembryonic group, which occurred at 116 kb, indicating the polyembryonic group has a shorter LD decay. This indicated moderate levels of LD with noticeable but not exceptionally strong SNP marker correlations in the populations. Overall, these analyses show that the population reported in these analyses is genetically diverse and is suitable for GWAS analyses.

### Genome-wide association study for polyembryony in mango

**Figure 3:**
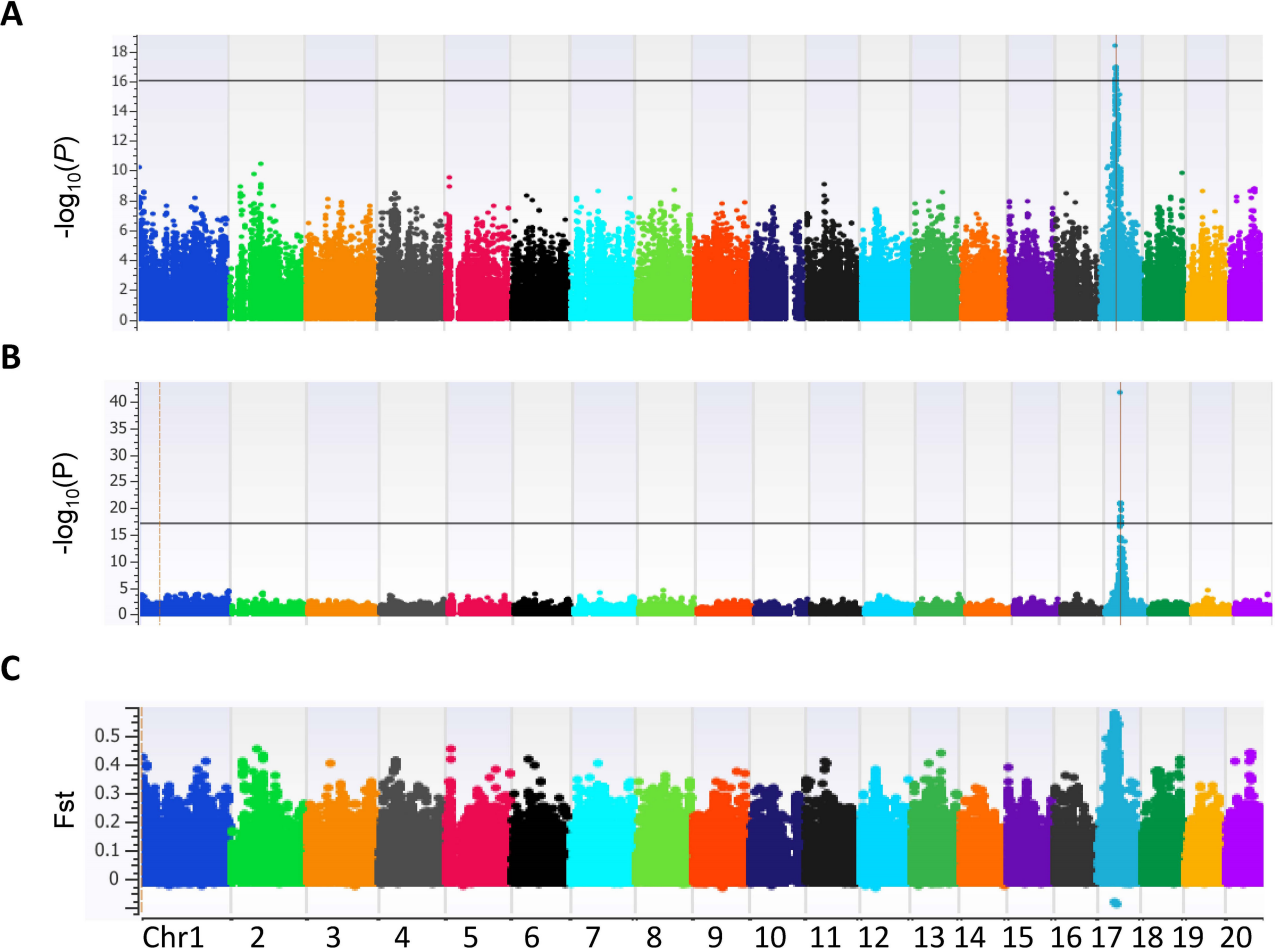
Manhattan plot illustrating single nucleotide polymorphism (SNP) marker-trait associations for polyembryony in 84 *Mangifera indica* accessions. The analysis utilized 133,590 SNPs across the genome, and associations were identified using two genome-wide association study (GWAS) models: **(A)** Mixed Linear Model (MLM): Highlights the significance of SNP associations with polyembryony, represented as −log10(P) values across 20 chromosomes. The threshold for significant associations is indicated by the horizontal line, showing distinct peaks on specific chromosomes. **(B)** Efficient Mixed-Model Association eXpedited (EMMAX): A complementary GWAS model providing additional insight into significant marker-trait associations, displayed as −log10(P) values. Peaks align with loci identified in the MLM analysis, validating consistent association signals. **(C)** Fixation Index (*Fst*): A plot showcasing the genetic differentiation between polyembryony-related and non-related accessions across the genome. Elevated *Fst* values indicate regions under potential selection, aligning with the association peaks identified in GWAS models.

**Figure 4:**
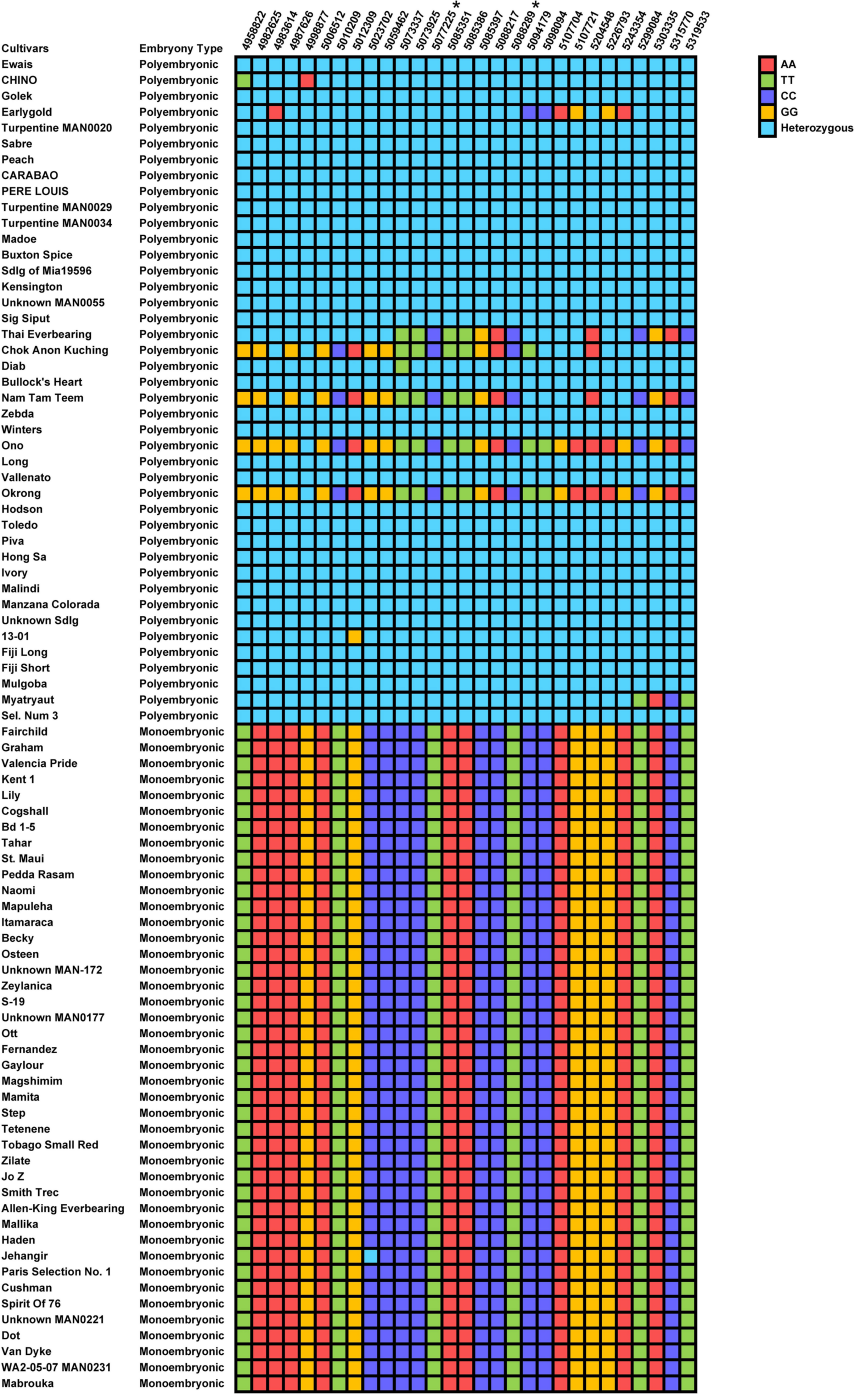
Nucleotide matrix displaying the distribution of single nucleotide polymorphism (SNP) alleles at 29 biallelic loci on chromosome NC_058153.1, which are strongly associated with polyembryony in *Mangifera indica*. The cultivars are organized based on their embryonic type, with polyembryonic cultivars listed at the top and monoembryonic cultivars at the bottom. Each cell represents the SNP allele observed for a specific cultivar at a given locus. Color Coding is as follows, Red (AA): Homozygous for allele A, Green (TT): Homozygous for allele T, Purple (CC): Homozygous for allele C, Orange (GG): Homozygous for allele G, Light blue (Heterozygous): Contains one allele from each variant (e.g., A:T, C:G). Markers with Asterisks: SNP loci specifically designed to capture genetic signals for polyembryony, highlighting key regions of genetic diversity.

Since polyembryony in mango is dichotomous and inherited dominantly, data were analyzed using the dominance genetic model using simple case/control binary classification of the data. *M. indica* chromosomes are numbered differently according to different genetic and genomic studies (Luo et al., 2016; Kuhn et al., 2017; Wang et al., 2020). For simplicity, we will be using the chromosome numbers according to the ‘Alphonso’ reference genome (GCA_011075055.1) (Wang et al., 2020). A prominent GWAS signal was detected on chromosome 17 (NC058153.1) using two different models, the mixed linear model (**Figure 3A**) and the EMMAX (**Figure 3B**). These results were also verified using additional models and variant data subsets presented in **Supplementary Figures S5**, **S6**. Statistical analyses delineated the associated chromosome area to a 360 kb haplotype block (NC_058153.1: 4958822 – 5319533). Within this block, 29 markers were determined to be significantly associated with polyembryony (p < 4.88 x E^-19^; FDR < 4.13 x E^-14^). Statistically significant markers were also confirmed using other commonly used test statistics for genome-wide association studies (**Supplementary Tables S2**, **S3**). Since population structure analyses revealed some cryptic relatedness, GWAS was performed using EMMAX, which accounts for kinship and data structure. These analyses essentially yielded similar results, suggesting that cryptic population structure in the data did not affect the occurrence of polyembryony (**Figure 3B**). A high fixation index (FST) of approximately 0.6 was also seen in this region (**Figure 3C**), indicating significant genetic distinctions between polyembryonic and monoembryonic cultivars. Call rate analyses showed that all these markers had a 100% call rate in all samples (**Supplementary Table S3**). MLM-based analysis, however, identified one marker (NC_058153.1_4998877_G_A) with twice as high a *p*-value as the rest of the markers. This marker is heterozygous (G/A) in all polyembryonic accessions except cultivar ‘Chino’ in which it was homozygous (A/A) and homozygous (G/G) in all monoembryonic accessions (**Figure 4**). These results show that dominant allele A is associated with polyembryony. Similarly, a majority of other polyembryony-associated markers were either homozygous or heterozygous, indicating that the polyembryonic locus is inherited dominantly (**Supplementary Table S4**), which is consistent with the genetic analysis finding that a single dominant gene controls polyembryony (Aron et al., 1998). Similarly, major/minor allele frequencies ranged from 0.71-0.75/0.29-0.25. Most of the 42 polyembryonic cultivars investigated in this study were heterozygous (**Supplementary Table S4**), shown in light-blue squares (**Figure 4**). Several of them, especially those from Southeast Asia such as ‘Okrong’, ‘Ono’, ‘Nam Tam Teem’ and ‘Chok Anon Kuching’ harbor homozygous alleles on most of these markers, indicating that the polyembryonic locus is fixed in these accessions. This is further supported by high LD values in the polyembryonic locus (**Supplementary Figure S7**).

### Analyses of genes in the polyembryony locus

Within the polyembryonic locus, 37 genes were located (**Table 1**). Annotations of these genes revealed that they belong to different functional groups, including transcription factors, cytokinin synthesis pathways, several different heat shock proteins, protein degradation, and calcium signaling (**Supplementary Table S5**). Among these genes, ‘Protein RKD4-like’ is the homolog of the *CitRKD1*, which has previously been shown to be associated with polyembryony in citrus (Shimada et al., 2018). In the reference genome of mango (GCA_011075055.1), this gene encodes a 117 amino acid long protein, which is 237 amino acids shorter than the *CitRKD1* (**Supplementary Figure S8**). This suggests that the annotation of *MiRKD4* gene may not be complete. For verification, the complete sequence of this gene and its isoforms is required through transcriptome studies.

**Table 1:** List of *Mangifera indica* genes within the LD block that is associated with polyembryony trait.

Gene Name	Transcript Name	Gene Product
LOC123200754	XM_044616122.1	Cytokinin dehydrogenase 7
LOC123201073	XM_044616524.1	Transcription factor MYB114-like
LOC123201086	XM_044616538.1	Transcription factor MYB1-like
LOC123200115	XM_044615245.1	Transcription factor MYB1-like
LOC123200786	XM_044616167.1	Transcription factor MYB123-like
LOC123200787	XM_044616168.1	NAD(P)H-quinone oxidoreductase subunit U, chloroplastic
TRNAD-GUC_18	unassigned_411	tRNA-Asp
LOC123200885	XM_044616265.1	Cadmium/zinc-transporting atpase HMA2-like
LOC123200788	XM_044616169.1	Peroxisome biogenesis factor 10
LOC123200999	XM_044616439.1	40S ribosomal protein S3-3
LOC123201000	XM_044616440.1	Thermospermine synthase ACAULIS5-like
LOC123200384	XM_044615553.1	Protein BIG GRAIN 1-like B
LOC123201048	XM_044616501.1	Omega-amidase, chloroplastic-like
LOC123199943	XM_044615000.1	Protein RKD4-like
LOC123200887	XM_044616269.1	Uncharacterized LOC123200887
LOC123200064	XM_044615162.1	PHD finger protein ALFIN-LIKE 4-like
LOC123201083	XM_044616534.1	Solute carrier family 25 member 44-like
LOC123200177	XM_044615319.1	17.1 kda class II heat shock protein-like
LOC123200161	XM_044615306.1	17.9 kda class II heat shock protein-like
LOC123199981	XM_044615043.1	17.3 kda class II heat shock protein-like
LOC123199983	XM_044615044.1	17.3 kda class II heat shock protein-like
LOC123200189	XM_044615336.1	17.3 kda class II heat shock protein-like
LOC123200930	XM_044616317.1	17.3 kda class II heat shock protein-like
LOC123200172	XM_044615315.1	17.1 kda class II heat shock protein-like
LOC123200068	XM_044615171.1	17.9 kda class II heat shock protein-like
LOC123201175	XM_044616651.1	AUGMIN subunit 1-like
LOC123201010	XM_044616454.1	Probable WRKY transcription factor 50
LOC123200613	XM_044615890.1	(S)-coclaurine N-methyltransferase-like
LOC123200614	XM_044615891.1	Uncharacterized protein At4g33100
LOC123200611	XM_044615882.1	Uncharacterized LOC123200611
LOC123200612	XM_044615889.1	Protein phosphatase 2C 53-like
LOC123200129	XM_044615263.1	NHP2-like protein 1
LOC123200699	XM_044616037.1	Serine/threonine-protein kinase tricornered-like
LOC123199882	XM_044614904.1	Protein ALP1-like
LOC123200373	XM_044615543.1	U-box domain-containing protein 35
LOC123201106	XM_044616566.1	Probable 2-oxoglutarate/Fe(II)-dependent dioxygenase
LOC123200956	XR_006498692.1	Uncharacterized protein

### Synteny analyses of the polyembryony locus

**Figure 5:**
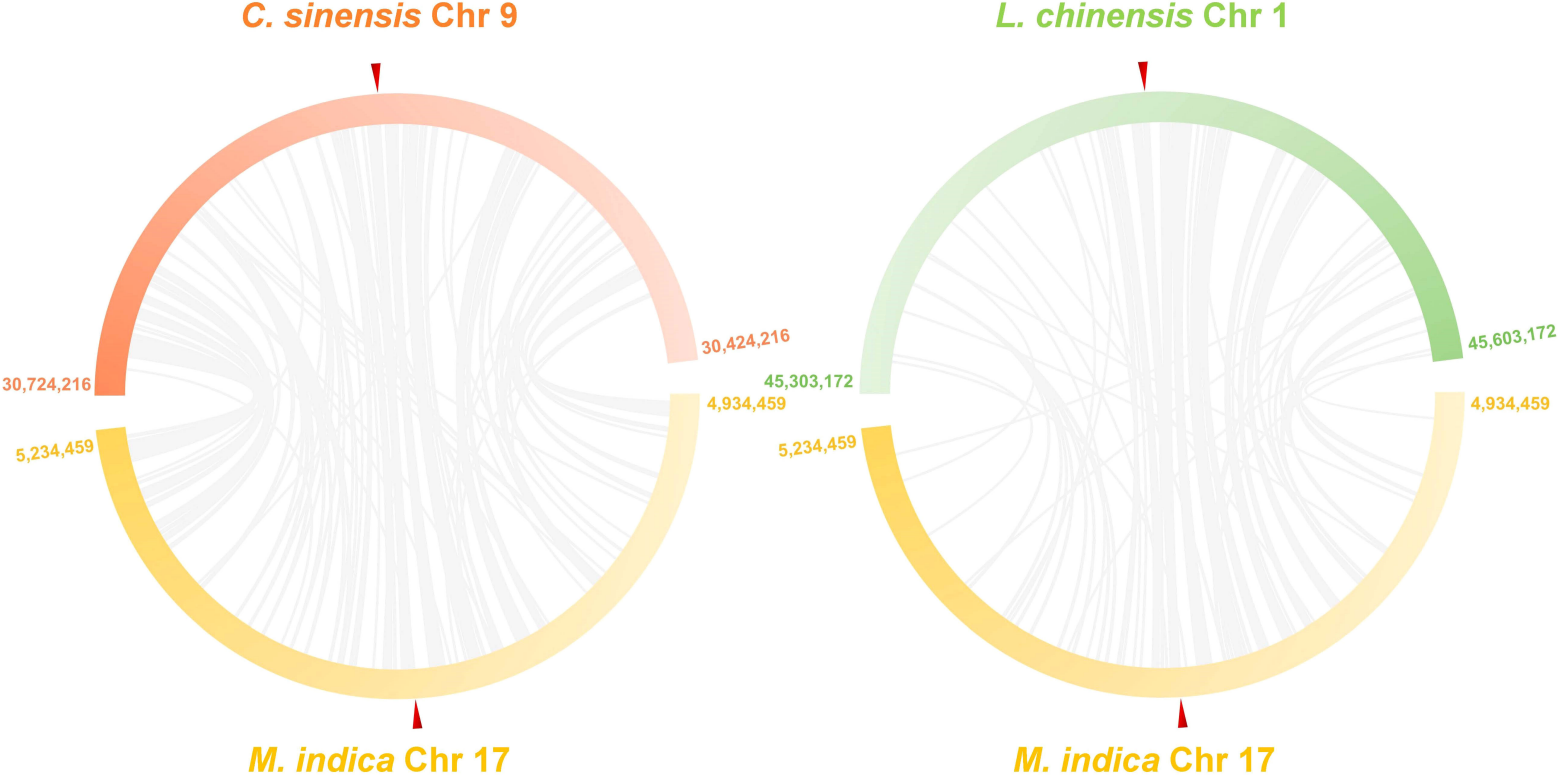
Comparative synteny plots of 300 kb regions centered on polyembryony genes in mango (GCA_011075055.1), citrus (GCF_022201045.2), and lychee (GCA_019925255.1). This synteny plot illustrates conserved genomic organization around the polyembryony gene in mango, citrus, and litchi. Each bar represents a chromosomal region, labeled with the chromosome number and positions. Ref triangles mark the positions of the *CitRWP* gene and its homologs. The figure was generated using Advanced Circos in TBtools 2.0 after aligning genomic sequences with blastn. Blocks with high synteny and collinearity indicate evolutionarily conserved regions across these fruit species.

It is predicted that citrus and mangos split from each other 76,000 years ago (MYA) (https://timetree.org/), suggesting that they might carry syntenic genomic regions, some of which might be harboring the polyembryony locus. To determine if the polyembryonic locus in citrus and mango are syntenic, we conducted synteny analysis between the citrus (GCF_022201045.2) and mango (GCA_011075055.1) reference genomes. These analyses revealed substantial synteny and collinearity around the polyembryony loci in citrus and mango (**Figure 5**, **Supplementary Figure S8**). *MiRKD4* was situated within a synteny block spanning 3,389 base pairs, and at least three other synteny blocks were observed to be closely and colinearly distributed in this region (**Supplementary Figure S8C**). Similarly, synteny at this locus was also observed in another closely related species, lychee, which is not polyembryonic (GCA_019925255.1), suggesting that despite the conservation of this locus in these fruit species, polyembryony is controlled by gene expression or other similar mechanisms.

### Development and validation of competitive allele-specific PCR Assays

To employ polyembryony-associated markers for marker-assisted selection of the polyembryony trait, two competitive allele-specific PCR-based genotyping assays were developed and validated: Kompetitive Allele-Specific PCR (KASP) and PCR Allele Competitive Extension (PACE). These assays are designed for convenient application in mango breeding programs. Five SNP markers (NC_058153_1_4987626_A_G, NC_058153_1_5077225_T_C, NC_058153_1_5088289_T_C, NC_058153_1_5012309_G_A, and NC_058153_1_5204548_G_A) associated with polyembryony were successfully developed into KASP and PACE assays. The assays were applied to 380 individual mango trees, including germplasm accessions, hybrids from breeding populations, and recently grafted accessions (**Supplementary Table S6**).

**Figure 6:**
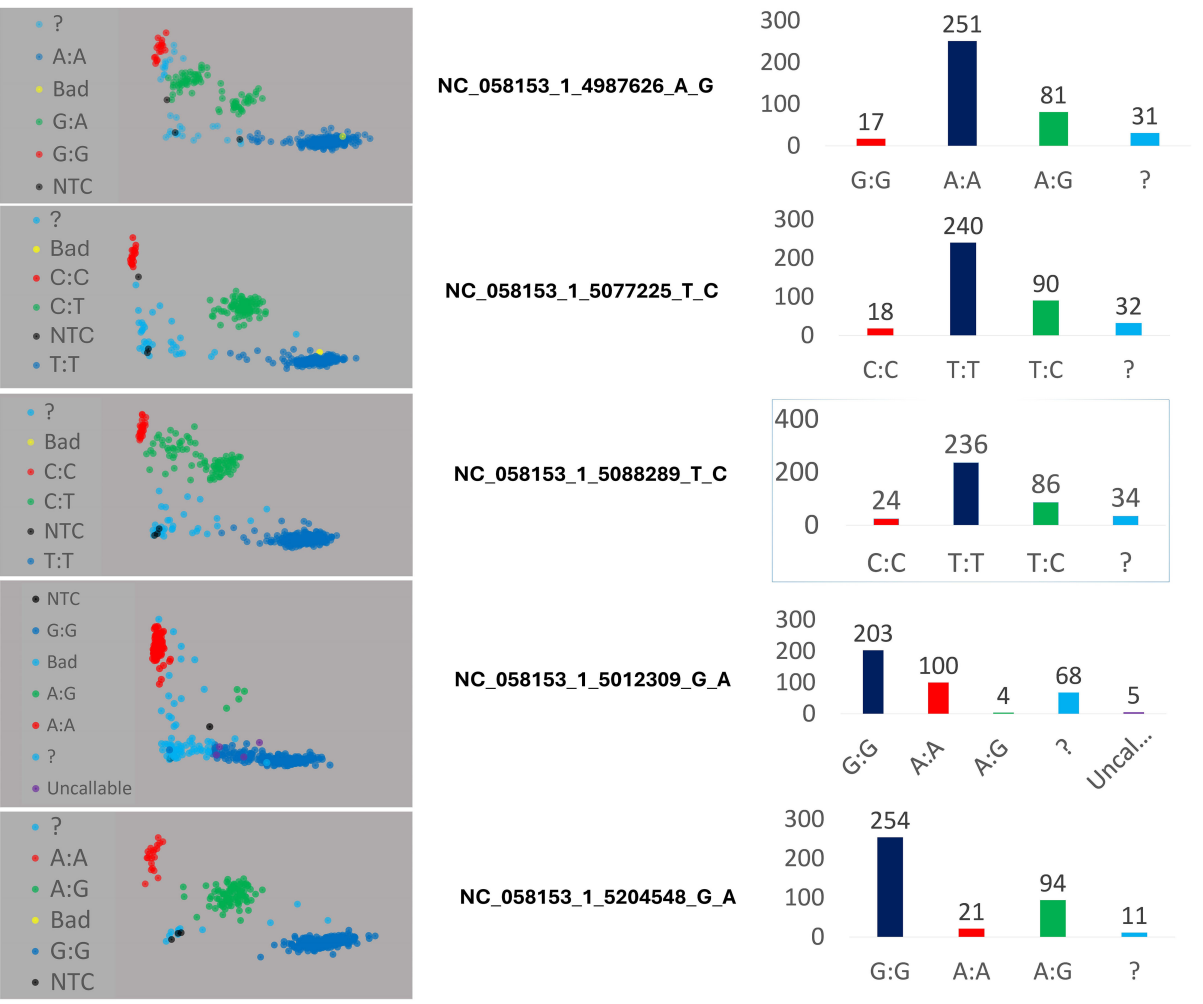
Scatter plots for five KASP markers across 380 *Mangifera indica* accessions, demonstrating clear genotypic clustering and allele distribution. The scatter plots highlight the accuracy of genotype calls and the distinct grouping of accessions into three genetic clusters. Blue and red dots represent homozygous allele and green dots heterozygous alleles. Black dots (NTC): No Template Controls, serving as negative controls for the assay, and ? (Undetermined) represent genotypes with ambiguous calls that could not be confidently assigned.

Validation results revealed that the KASP genotype calls were consistent with the original SNPs obtained from Illumina whole genome resequencing data. Furthermore, genotyping data for each KASP marker across four 96-well plates were visualized using SNPviewer software (see **Supplementary Figure S9**). Scatter plots of all markers demonstrated the accurate distribution of the mango accessions across three distinct clusters (refer to **Figure 6**). In the scatter plots, red dots denoted homozygous alleles (HEX- polyembryony allele), blue dots represented homozygous monembryony alleles (FAM), and green dots denoted heterozygous alleles. Additionally, unidentified accessions were depicted as light blue dots, while black dots represented no call outcomes. Two of the five KASP markers mentioned earlier (NC_058153_1_5077225_T_C and NC_058153_1_5088289_T_C), flanking the *MiRKD4* gene, were employed and verified using the PACE assay kit on a randomly selected subset of 47 accessions. The allocation of these 47 mango accessions into mono- and polyembryonic types, as determined by PACE assays, is depicted in **Supplementary Figure S10**. In comparison to DNA-seq-based and KASP-based SNP calling, 42 out of the 47 accessions were accurately identified by PACE using both SNPs. The single exception, ‘Early Gold’, was the only sample unidentifiable by one PACE SNP and was accurately identified by the other PACE SNP. Consequently, the combined utilization of both PACE markers effectively validated all DNA-seq-based and KASP-based determinations. These findings underscore the capability of these two markers to discern polyembryony with near-perfect certainty.

### Validation and practical application of the single nucleotide polymorphism genotyping assays associated with polyembryony in a breeding population

The practicality and applicability of the KASP markers were validated by analyzing 34 recently grafted accessions (refer to **Supplementary Table S6**). This scrutiny confirmed that the grafted trees possessed the identical alleles as the original mother tree. Notably, the polyembryony allele was inherited identically in grafted accessions bearing the IDs MAN0362 and MAN0363, arising from accessions MAN0005 and MAN0007, respectively. This attests to the significant utility of the KASP markers in identifying the polyembryony allele at the seedling stage. Furthermore, upon analyzing the open-pollinated trees in our mango samples, we could ascertain the alleles’ lineage. Specifically, among the seedlings of the ‘Tommy Atkins’ cultivar, three open-pollinated trees, represented by the IDs MAN0318, MAN0339 and MAN0350, exhibited heterozygous polyembryonic alleles, even though the ‘Tommy Atkins’ variety, represented by ID MAN0003, is monoembryonic. Likewise, two open-pollinated trees, represented by the IDs MAN0253, MAN0263, MAN0264, and MAN0330, and by MAN0315, originating from the ‘Keitt’ cultivar, manifested heterozygous polyembryonic alleles, contrary to the monoembryonic nature of ‘Keitt’. The remaining open-pollinated seedlings from these two mother cultivars were confirmed to carry the monoembryonic alleles (refer to **Supplementary Table S6**). These outcomes affirm the practical application of the SNP genotyping assays developed in this study.

Upon evaluation of all 47 tested mango accessions, it was observed that specific accessions in the study were either inaccurately called or not called at all by three distinct SNP genotyping assays, namely whole genome sequencing data, KASP, and PACE assays (**Supplementary Table S7**). A comparative analysis of the three types of call was conducted to rectify any mislabeling observed in the genotyping assays and ensure the accuracy of phenotypic data, as well as to make precise predictions regarding the type of polyembryony in each accession (**Table 2**). For instance, the accession MAN0005_Ewais exhibited a heterozygous allele in all types of SNP calling for both markers, except for the PACE assay marker NC_058153_1_5088289_T_C, which was not called. Considering the polyembryony phenotype of this accession, it is concluded that it possesses a heterozygous NC_058153_1_5088289_T_C SNP. Additionally, all accessions of TURPENTINE and Sabre demonstrated the presence of a heterozygous allele in all three SNP assays for both markers, except for the PACE marker NC_058153_1_5088289_T_C, which was indicated as (T:T). Therefore, it is appropriate to rectify these calls in the TURPENTINE and Sabre accessions to (C:T), a correction that is substantiated by the phenotypic data. In the case of accession MAN0046 SDLG OF MIA-06895, which was classified as monoembryonic and demonstrated a homozygous allele (T:T) in all SNP callings, with the exception of the DNA sequencing data, where it was labeled as missing (N) for NC_058153_1_5077225_T_C, revision of the miscalled (N) to (T:T) is warranted based on the phenotypic data and all three genotyping calls. Furthermore, for two different accessions, MAN0010 Diplomatico and MAN0043 O.P. Sandersha, with missing information on the type of embryony, SNP calling suggests that Diplomatico is polyembryonic, whereas O.P. Sandersha is monoembryonic.

**Table 2:** The validation and correction of miscalling and mislabeling data in different mango accessions using DNA sequencing, KASP and PACE calling in two markers.

Mango Code	Cultivar Name	NC_058153_1_5077225_T_C			NC_058153_1_5088289_T_C			Embryony Types	
		DNA	LGC	PACE	DNA	LGC	PACE	Original	Corrected
		Miscalling							
MAN0005	Ewais	C:T	C:T	C:T	C:T	C:T	? **/C:T**	P	P
MAN0006	TURPENTINE_166	C:T	C:T	C:T	C:T	C:T	T:T/**C:T**	P	P
MAN0018	Earlygold	C:T	C:T	? **/C:T**	C:T	C:T	? **/C:T**	P	P
MAN0020	TURPENTINE	C:T	C:T	C:T	C:T	C:T	T:T/**C:T**	P	P
MAN0021	Sabre	C:T	C:T	C:T	C:T	C:T	T:T/**C:T**	P	P
MAN0026	PERE LOUIS	C:T	C:T	?/**C:T**	C:T	C:T	C:T	P	P
MAN0046	SDLG OF MIA-06895	N/**T:T**	T:T	T:T	T:T	T:T	T:T	M	M
Mislabeling									
MAN0010	Diplomatico	C:T	C:T	C:T	C:T	C:T	C:T	?	**P**
MAN0038	Sandersha	T:T	T:T	T:T	T:T	T:T	T:T	M	**M**
MAN0043	O.P Sandersha	T:T	T:T	T:T	T:T	T:T	T:T	?	**M**

The call with bold and underline text refer to the correction after comparison with the other calling methods.

## Discussion

### Geographical distribution and population structure of mango cultivars

*M. indica* is native to South Asia, specifically to India, Bangladesh, and Myanmar. Over the centuries, mango cultivation has spread to various tropical and subtropical regions worldwide. Today, it is grown in many countries with suitable climates, including Southeast Asia, Africa, the Middle East, South America, and parts of the United States. Crop genetic diversity patterns can provide information about their history of domestication. The model-based analysis of clustering grouped the mango cultivars into three major groups. The ΔK value of Evanno’s algorithm predicted K = 3 is true by differentiating the mango cultivars as per their geographic origin, i.e., Southeast Asian region, India, and US and different countries. So, in this study, three main groups were identified by our analysis of the genetic structure of cultivated mango germplasm: the Indian cluster, which contains the monoembryonic varieties; southeast Asian cultivars, which contain the polyembryonic varieties; and an admixture cluster representing US, Caribbean, and different countries admixed with polyembryony and monoembryony. The difference in polyembryony between Indian and Southeast Asian cultivars confirms the classification of mango cultivar types as Indian or Indochinese, as per traditional standards (Campbell and Sauls, 1994; Warschefsky and von Wettberg, 2019), as well as earlier genetic analyses of mango germplasm diversity (Schnell et al., 2006; Dillon et al., 2013; Warschefsky and von Wettberg, 2019; Wilkinson et al., 2022). Furthermore, the variations between cultivars in the US, the Caribbean, and other countries align with results from a recent study on the diversity of mango germplasm, which differentiated between cultivars in Asia and the different regions (Sherman et al., 2015; Warschefsky and von Wettberg, 2019). In addition to the three cultivars groups, the principal components analysis shows two major clusters in the population structure. The admixture cluster 3 is divided in the phylogenetic tree into two subclusters displaying polyembryony cultivars. The same results were reported in (Dillon et al., 2013; Warschefsky and von Wettberg, 2019; Yamanaka et al., 2019; Wilkinson et al., 2022). Understanding population structure is crucial in genetic studies because if not adequately accounted for, it can lead to spurious associations in GWAS. Subpopulations may have differences in allele frequencies unrelated to the trait of interest but can confound GWAS results. So, statistical methods, such as principal component analysis (PCA) or kinship matrix, are employed to correct for population structure in GWAS. Accounting for population-specific effects increases the accuracy of identifying associations relevant to specific groups.

### Genome-wide association studies for polyembryony

With the release and public availability of mango genome sequences (Wang et al., 2020) and the development of SNPs, genome-wide association studies can be carried out to identify alleles and chromosomal regions associated with horticulturally important traits. Here, we report a pioneering GWAS applied to a genetically and phenotypically diverse collection of *M. indica* to identify markers associated with polyembryony. In the present study, we identified a strong signal on chromosome 17 (NC058153.1). Previously, using a limited number of SNPs, (Kuhn et al. 2019), identified several markers associated with polyembryony in different linkage groups, including LG4, 8,13,15,17, and 19, some of which were in the same relative positions in the same linkage group (LG) of two different mapping populations that shared a single maternal polyembryonic parent (‘Kensington Pride’). Interestingly, we found markers associated with polyembryony in a 360 kb haplotype block on chromosome 17 (NC_058153.1: 4958822- 5319533). Similar results have been reported in citrus where a quantitative trait locus associated with polyembryony is located on *Citrus* LG1 in both *Satsuma mandarin* and *Ponkan mandarin*, and the genomic region containing the polyembryony locus spans approximately 380 kb (Nakano et al., 2008; Nakano et al., 2012). The analysis of LD provides important information on the markers that tend to be inherited from generation to generation. According to Aron et al (Aron et al., 1998), polyembryony is a dominantly inherited trait controlled by a single locus (Kuhn et al., 2017). In support of this, we discovered that all markers in the 360 kb haplotype block on *M. indica* chromosome 17 were either heterozygous or homozygous. The major/minor allele frequencies were 0.71-0.75/0.29-0.25. For example, SNP marker NC_058153.1_4998877_G_A is heterozygous (G/A) in all polyembryonic accessions except cultivar ‘Chino’ in which it was homozygous (A/A) and homozygous (G/G) in all monoembryonic accessions. These findings demonstrate the relationship between polyembryony and dominant allele A. Kuhn et al (Kuhn et al., 2017), reported that the polyembryony locus is located in a single locus on LG 8 in two of the three mapping populations and confirmed that the locus regulating polyembryony is heterozygous with a dominant polyembryony allele, whereas monoembryonic individuals were homozygous recessive.

Gene annotation of the 360 kb haplotype block, which contains 29 significant markers, revealed 37 genes, which are associated with different physiological and molecular functions such as transcription, protein turnover, signaling and growth regulators. Whether these genes play a role in polyembryony will require functional characterization in future studies. Interestingly, this block harbors a gene *MiRKD* (LOC123199943), which encoded an RKD4-like protein and has been shown to play a role in polyembryony in citrus. The citrus homolog of *MiRWP/MiRKD4* protein, which has the RWP-RK domain, has been shown to regulate the expression of genes related to egg cell development (Schauser et al., 2005). In citrus, *CitRWP* has been suggested as the causal gene for polyembryony (Wang et al., 2017). In GWAS of a citrus F2 population segregating for the polyembryony trait and more than 100 citrus accessions consisting of apomictic and non-apomictic types, identified *CitRWP* as a candidate gene causing polyembryony. They suggested that the *CitRWP* gene was overexpressed due to the insertion of a miniature inverted-repeat transposable element (MITE) in the promoter region and that polyembryony resulted from this abnormal expression. The RKD gene (*CitRKD1*) was reported to regulate somatic embryogenesis because its transcription level in polyembryonic citrus varieties was higher than in monoembryonic varieties and the insertion of MITE sequence in the promoter region of *CitRWP* gene was only seen in polyembryonic citrus varieties (Schauser et al., 2005; Wang et al., 2017; Shimada et al., 2018). In *Arabidopsis* an analysis of a segregation population revealed that the MITE co-segregated with poly/monoembryonic type seeds (Schauser et al., 2005). The RKD genes are also suggested to regulate a gene expression program related to egg cells in *Arabidopsis* and citrus. MITEs are small DNA transposons (between 100 and 600 bp) lacking genes that code for transposase (Shimada et al., 2018; Jeong et al., 2023). MITEs are relatively long-homogeneous DNA transposons in contrast to other non-autonomous DNA transposons. Phylogenetic analyses of diverse MITEs in citrus suggest that more progenitors may have been amplified to produce such great length-homogeneity (Jeong et al., 2023). It would be interesting to conduct similar studies on an extensive collection of poly and mono-embryonic mango accessions and investigate whether the types and patterns of MITE in these citrus and Mangos are identical or have evolved independently.

Synteny analyses of the polyembryony loci between *M. indica* and *Citrus* and *M. Indica* and *Litchi* showed extensive microsynteny regions, suggesting that this genomic locus and apomixis in these species have remained conserved. Targeted synteny analyses also revealed collinearity among the citrus and mango *RKD4* genes, especially in the exon regions, further supporting that apomixis in these two species is most likely controlled by this gene.

### Significance and conclusions

Polyembryony is important for clonally propagating elite cultivars through seeds, particularly in fruit trees with distinct and highly heterozygous fixed genotypes. Using GWAS, we identified markers associated with the polyembryony trait in Mangos. Understanding the genetic mechanism of polyembryony promises to accelerate the breeding of Mangos and opens the possibility of applying this technology to other fruit tree crops using biotechnological approaches.

In conclusion, we have mapped the polyembryony locus in Mangos. Implementing marker-assisted selection for polyembryony at the early seedling stage will substantially reduce operational costs associated with maintaining breeding populations of mango trees over multiple years and locations. Other applications of findings in this report include expanding polyembryony to other fruit crops in the anacardiaceous family and beyond. Based on comparative GWAS studies in citrus as well as functionally analyzing the polyembryony-associated *RKD1* gene through antisense repression, it is highly likely that the mango homolog of *RKD1* is involved in controlling polyembryony in mango. However, understanding the biological significance of how RKD1 mechanistically affects polyembryony will require gain-of-function and loss-of-function experiments using CRISPR or other appropriate approaches, such as gene silencing.

## Materials and methods

### Plant material

The United States Department of Agriculture, Agricultural Research Service, Subtropical Horticulture Research Station, Miami, FL maintains a diverse collection of 278 *M. indica* germplasm, originating from different regions of the world. A set of 84 *M. indica* accessions consisting of an equal number of polyembryonic and monoembryonic accessions were used in this study for whole genome resequencing. A list of all *Mangifera* spp. accessions used in this study is provided in **Supplementary Table S1**.

### Embryony determination

Embryony was determined in mango cultivars as part of a fruit phenotype data collection over multiple years. Each year, at least twelve ripe fruits were collected from each cultivar. The seed was extracted from the husk, and the seed coat was removed if needed. Seeds of multiple embryos were recorded as polyembryonic, and those with a single embryo as monoembryonic. Percent polyembryony in a cultivar was determined by comparing the number of fruits that showed polyembryony with the total number of fruits collected.

### Whole genome sequencing and single nucleotide polymorphism calling

Using a proprietary protocol, DNA was isolated from leaf samples of all mango accessions by Biosearch Technologies, LGC (Middleton, WI). DNA quality was assessed with a Quant-iT™ dsDNA Assay Kit, and library preparation included shearing 500 ng of gDNA with a Covaris^®^ LE220 Focused Ultrasonicator, followed by normalization for shotgun fragment library creation using Biosearch Technologies NxSeq^®^ UltraLow and NxSeq HT Dual Indexing Kit. Libraries were sequenced using the Illumina 150x PE platform at the University of Wisconsin Biotechnology Center on an Illumina NovaSeq™ 6000 instrument. Reads were quality trimmed, and the adaptor was clipped simultaneously using a 10 bp sliding window with a mean Q score of 20 and a minimum final length of 50 bp. Cleaned reads were aligned to the Alphonso reference genome CATAS_Mindica_2.1 (GCA_011075055.1) (Wang et al., 2020) using BWA-mem using default parameters. The GATK Picard Tools were used to mark duplicates with default parameter settings.

SNPs were called using DeepVariant v1.4.0 (Poplin et al., 2018) with –model type=WGS for each sample separately using a Singularity container built from a pre-built docker container downloaded from the public repository (https://github.com/google/deepvariant). The resulting gVCF files were combined into a single cohort using GLnexus v1.2.7. with –config DeepVariantWGS for Whole Genome Sequencing. GLnexus was run from a Singularity container built from a docker container (https://github.com/dnanexus-rnd/GLnexus). SNPs were filtered using vcftools with the following parameters: minor allele frequency=0.05, quality≥30, missing call rate=0.9, MIN_DEPTH=6 and MAX_DEPTH=1000. This resulted in 134,251 high-quality SNPs (MAF > 0.05, missing call rate < 0.1, Median gap=0.306kb, average gap=2.645kb). The SNP distribution density on chromosomes was visualized using CMplot (Yin et al., 2021) and custom *R* code based on the Alphonso reference genome (GCA_011075055.1) CATAS_Mindica_2.1 (Wang et al., 2020).

### Linkage disequilibrium

Linkage disequilibrium (LD) among all markers was estimated using TASSEL 5 (Bradbury et al., 2007) through pairwise LD, which was evaluated using squared band-frequency correlations (*r* ^2^). To reduce the influence of a strong LD on the assessment of population stratification, a subset of 18,823 SNPs was selected by setting an LD (*r^2^*) threshold to 0.5 for the SNP pairs in a sliding window of 50 SNPs using SNP and Variation Suite (SVS) v8 (Golden Helix, Inc., Bozeman, MT, www.goldenhelix.com). Degree of linkage disequilibrium (LD) was estimated using the squared allele-frequency (*r^2^*) based on pair-wise comparison of all 137,981 SNP markers by PopLDdecay v3.42 (Zhang et al., 2019). A plot of the LD estimates vs physical distance (Kb) indicating LD decay in all chromosomes was drawn by the Plot_MutiPop.pl script provided in the software.

### Analysis of population structure

In order to determine population structure within the 84 accessions, K-means clustering was conducted in the Bayesian software STRUCTURE v.2.3.4 (Pritchard et al., 2000). The STRUCTURE is a Bayesian Markov chain Monte Carlo (MCMC) program that assigns individuals into genetic clusters (K) based on their genotypes by assuming Hardy Weinberg equilibrium (HWE) within a cluster. It gives each accession an admixture coefficient to depict the proportion of the genome originating from a particular K cluster. We ran the admixture model and the correlated allele frequency model 75 with ten independent runs of 100,000 burn-in and 100,000 MCMC iterations for K = 1 -10. We inspected summary statistics of MCMC runs to ensure convergence of model parameters. The optimal value of K was determined using StructureHarvester v.0.6.94 (Earl and vonHoldt, 2011) according to the ΔK method as reported (Evanno et al., 2005). Additionally, principal component analysis (PCA) was used to visualize and confirm the population structure within the dataset of 84 mango cultivars in the R/adegenet package v.2.1.10 (Jombart, 2008). The genetic distance between markers was calculated using a simple matching coefficient using R-package ‘ade4’ (Dray et al., 2023). For phylogenetic analysis, 18,823 SNPs were aligned using MUSCLE (Edgar, 2004). The alignment file was used to build a consensus phylogenetic tree using the Tamura-Nei genetic distance model (Tamura and Nei, 1993) and the unweighted pair group method with arithmetic mean dendrogram analysis (UPGMA) tree build method. All three analyses were run with an LD pruned set of 18,823 SNP that are not in linkage disequilibrium (LD).

### Genome-wide association studies

The GWAS was performed using the software SNP and Variation Suite (SVS) v8 (Golden Helix, Inc., Bozeman, MT, www.goldenhelix.com) using a mixed linear model (MLM) and EMMAX (Efficient Mixed Model Association Expedited) (Kang et al., 2010), which allows accounting for population structure and any cryptic relatedness. Manhattan plots were created using the −log10 *p* values. To adjust for any cryptic relatedness, a kinship matrix based on identity-by-state (IBS) alleles that are the same irrespective of their ancestral origin was used in these analyses. IBS was calculated using the Golden Helix SVS suite. The results were further validated through two additional models: a general linear model (GLM) with principal component analysis (PCA) and a mixed linear model (MLM) incorporating both PCA and kinship matrix (*K*), with a strict Bonferroni Correction threshold -Log10(0.05/n), where n is the adequate number of independent SNPs. The analysis was conducted using TASSEL 5, and the output was visualized using CMplot.

### Synteny analyses of the polyembryony locus

The synteny analyses of the polyembryony locus between citrus and other fruit species were conducted using *M. indica* Alphonso reference genome (GCA_011075055.1) against *Citrus sinensis* reference genome (GCF_022201045.2) and *Litchi chinensis* reference genome (GCA_019925255.1) (Hu et al., 2022) separately. The genome sequences were aligned by blastn, and the results were visualized using Advanced Circos in TBtools 2.0 (Chen et al., 2020).

### Development of competitive allele-specific PCR assays

Our goal in this analysis was to screen all our mango accessions using KASP and PACE assays for significant loci to confirm the validity of the markers found in this study. Five important markers were selected for this study and converted to KASP markers: NC_058153_1_4987626_A_G, NC_058153_1_5077225_T_C, NC_058153_1_5088289_T_C, NC_058153_1_5012309_G_A, and NC_058153_1_5204548_G_A. To extract the DNA and run the KASP markers for the chosen five markers, 380 mango trees’ leaves were sampled and sent to Biosearch Technologies, LGC (Middleton, WI), where DNA was isolated and KASP genotyping assays were performed. SNP calling data of the four 96-well plates were visualized using the SNPviewer software (LGC, Biosearch Technologies, Beverly, MA, USA) to analyze the SNP allele call data for individual markers based on the fluorescence signal. The target polyembryony allele was labeled with HEX (red), the polyembryony allele was labeled with FAM (blue), and the heterozygous polyembryony allele was labeled with green color.

### Validation of selected data set using PCR allele competitive extension

The PACE is a fluorescent competitive allele-specific PCR genotyping assay that we used to test and validate our phenotypic poly and monoembryony results for a set of 47 mango accessions. Two KASP markers (NC_058153_1_5077225_T_C and NC_058153_1_5088289_T_C) from the five assayed by LGC were used in the analysis. The DNA concentrations of 47 samples were diluted to 50 ng/µL following the procedures provided in the LGC Genomics manual for KASP genotyping. For each sample, PACE assays were conducted in a 10 µL reaction consisting of 2.5 µL of DNA, 2.5 µL KASP assay mix, which included 0.165 µL of each primer and 0.41 of standard reverse primer, and 5 µL of 2x PACE 2.0 Genotyping master mix (3CR Bioscience, UK). Conditions for thermal cycling were as follows: Step 1. 94°C for 15 minutes. Step 2. 10 cycles of touchdown PCR: 94°C for 20 seconds followed by 65–57°C for 60 seconds with a 0.8°C decline per cycle. Step 3. 26 cycles: 20 seconds at 94°C and 60 seconds at 55°C. The PCR reactions were run and analyzed for SNP calling in The Applied Biosystems QuantStudio Dx Real-Time PCR. Data was visualized in Excel.

## Data Availability

The datasets presented in this study can be found in online repositories. The names of the repository/repositories and accession number(s) can be found in the article/**Supplementary Material**.
