# Supplementary material for: GWAS identifies a polyembryony locus in mango: development of KASP and PACE markers for marker-assisted breeding
Source: Front Plant Sci. 2025 Jan 29;16:1508027. doi: 10.3389/fpls.2025.1508027 (PMC11814187; doi:10.3389/fpls.2025.1508027)
Supplement: Supplementary file 1 [file DataSheet1.pdf]

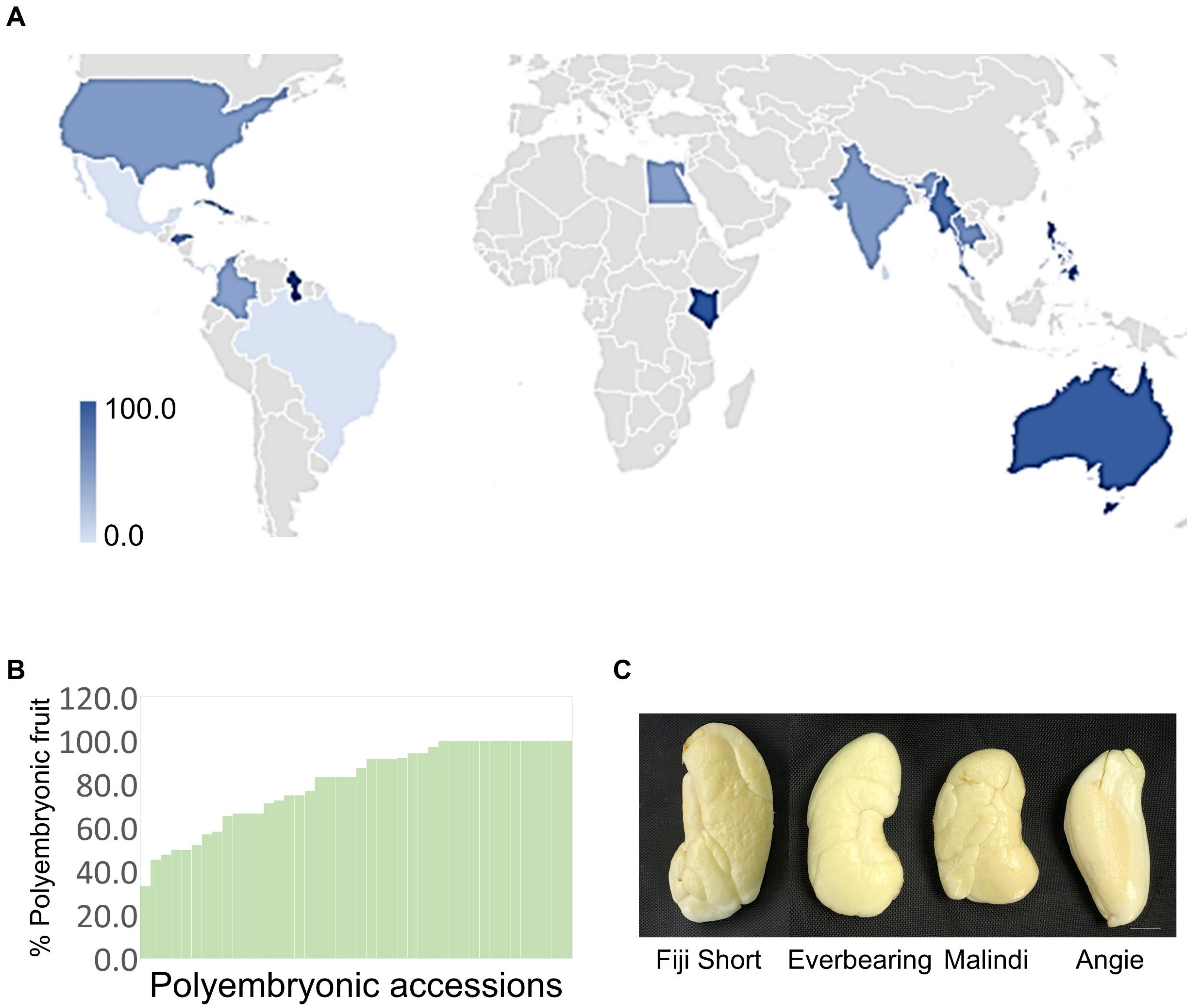

Figure S1: Geographical distribution of polyembryonic and monoembryonic *M. indica* accessions used in this study. (A) Blue shading shows frequency of polyembryonic fruit with dark blue corresponding to 100% polyembryonic fruit and light blue to monoembryonic fruit. (B) Distribution of proportion of polyembryonic fruit in each polyembryonic accession. Frequency of polyembryonic fruit ranged from 33 - 100% in polyembryonic cultivars. (C) Representative pictures of polyembryonic (Cvs. ‘Fiji Short’, ‘Everbearing’, and ‘Malindi’) and monoembryonic (Cv. ‘Angie’) mango seeds.

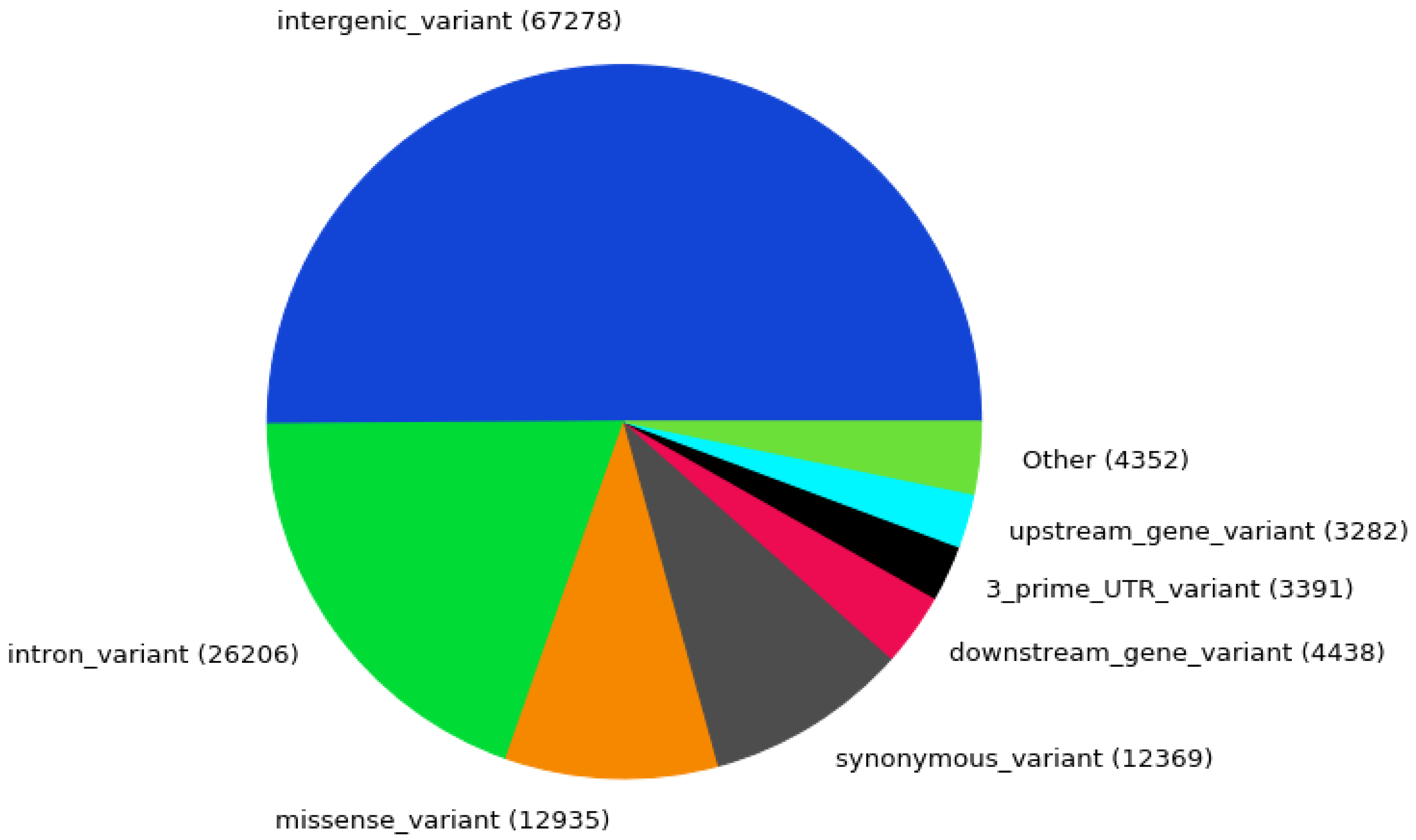

Figure S2: Statistical analysis of the annotation result of single nucleotide polymorphism (SNP) used in this study.

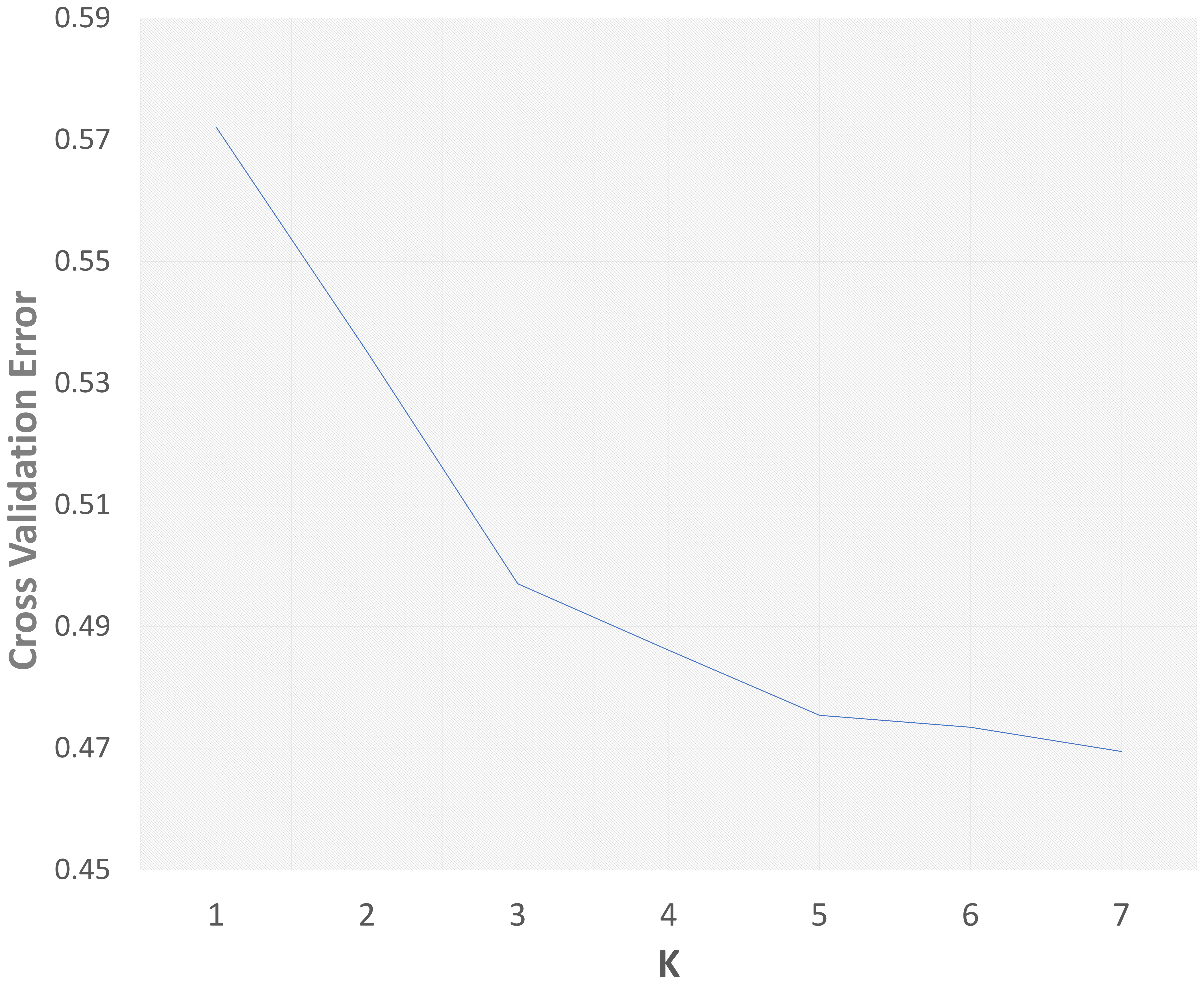

Figure S3: ADMIXTURE cross-validation error plot from K=1 to K=7. When K is greater than 3, the trend tends to be flat with no significant differences shown.

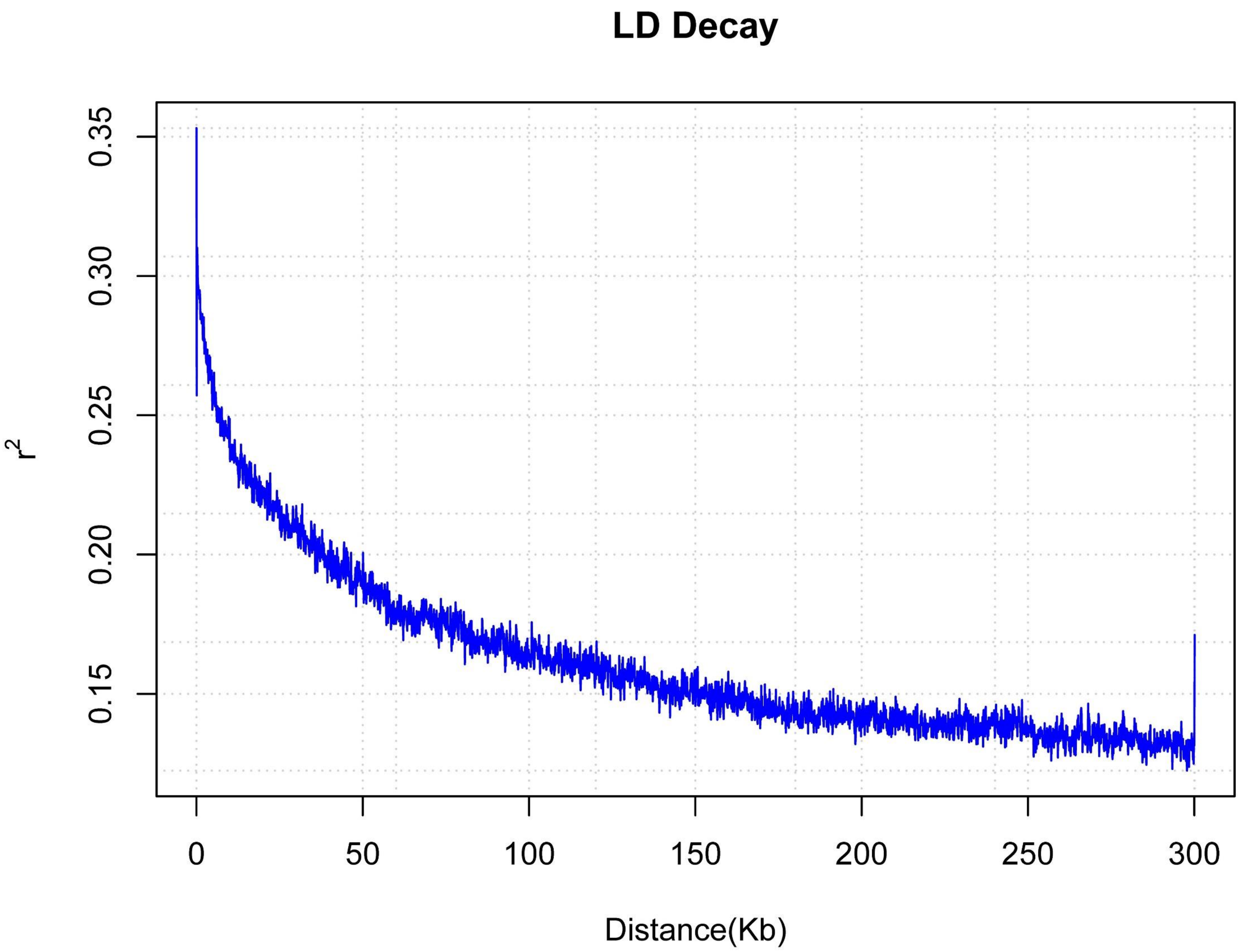

Supplementary Figure S4: Linkage disequilibrium (LD) decay plot using PopLDdecay showing genomic distance versus r^2 between pairs of the 137K SNPs within each 100 bp window.

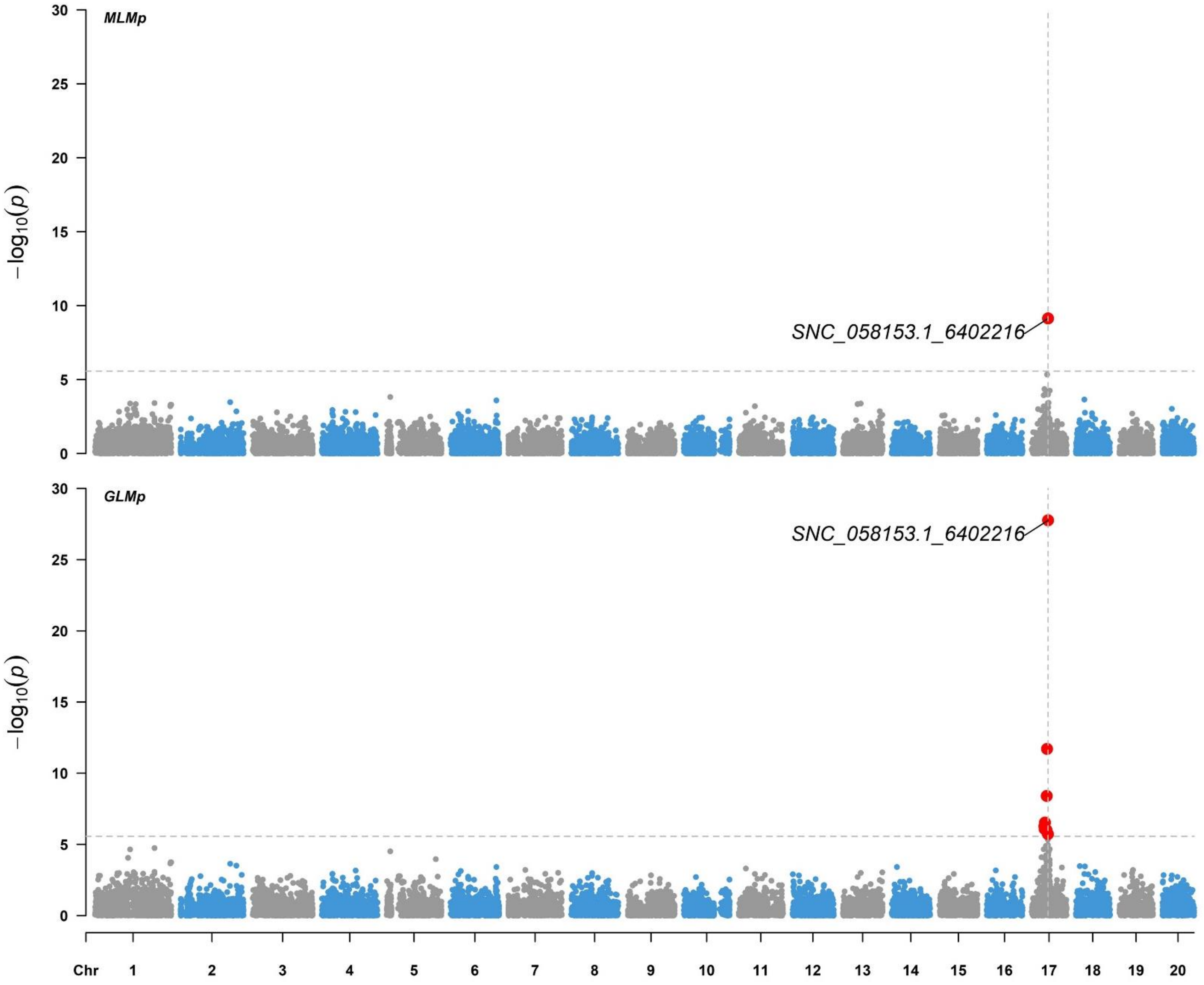

Supplementary Figure S5: Vertical alignment of Manhattan plots comparing the results of mango polyembryony GWAS analyses on different models. Upper panel: MLM (PCA + K) model. Lower panel: GLM (PCA) model. The X-axis represents the 20 chromosomes of the Mango reference genome CATAS_Mindica_2.1. Significant signals from a strict Bonferroni threshold of 0.05/(number of SNPs) are highlighted in red. Markers supported by both models are labeled with the name.

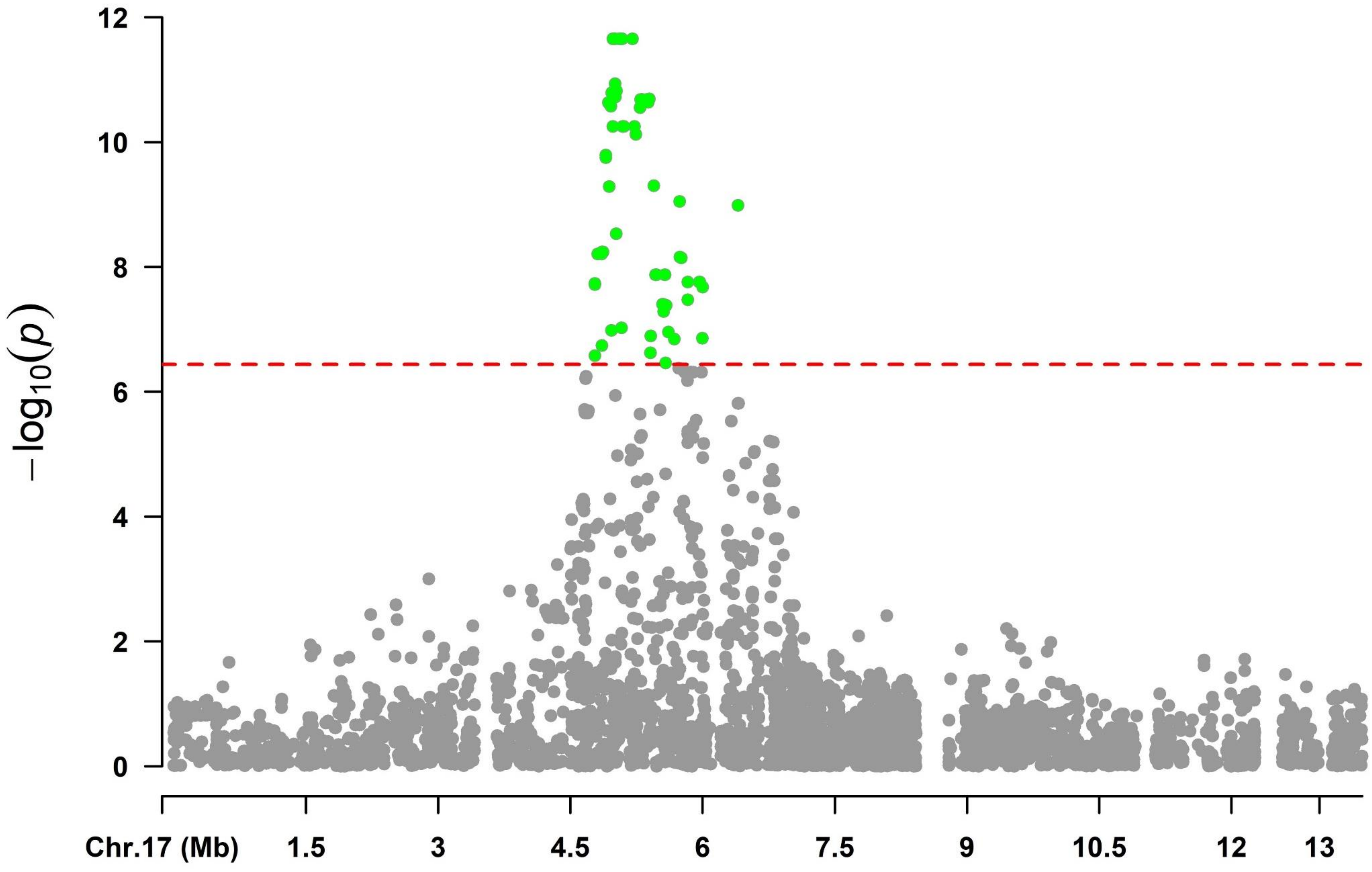

Supplementary Figure S6: Single-chromosome Manhattan plot showing the association between SNPs on Chr 17 and polyembryony in *Mangifera indica*. The GWAS analysis was performed based on 137k SNP data and used MLM (PCA + K) model in Tassel 5.

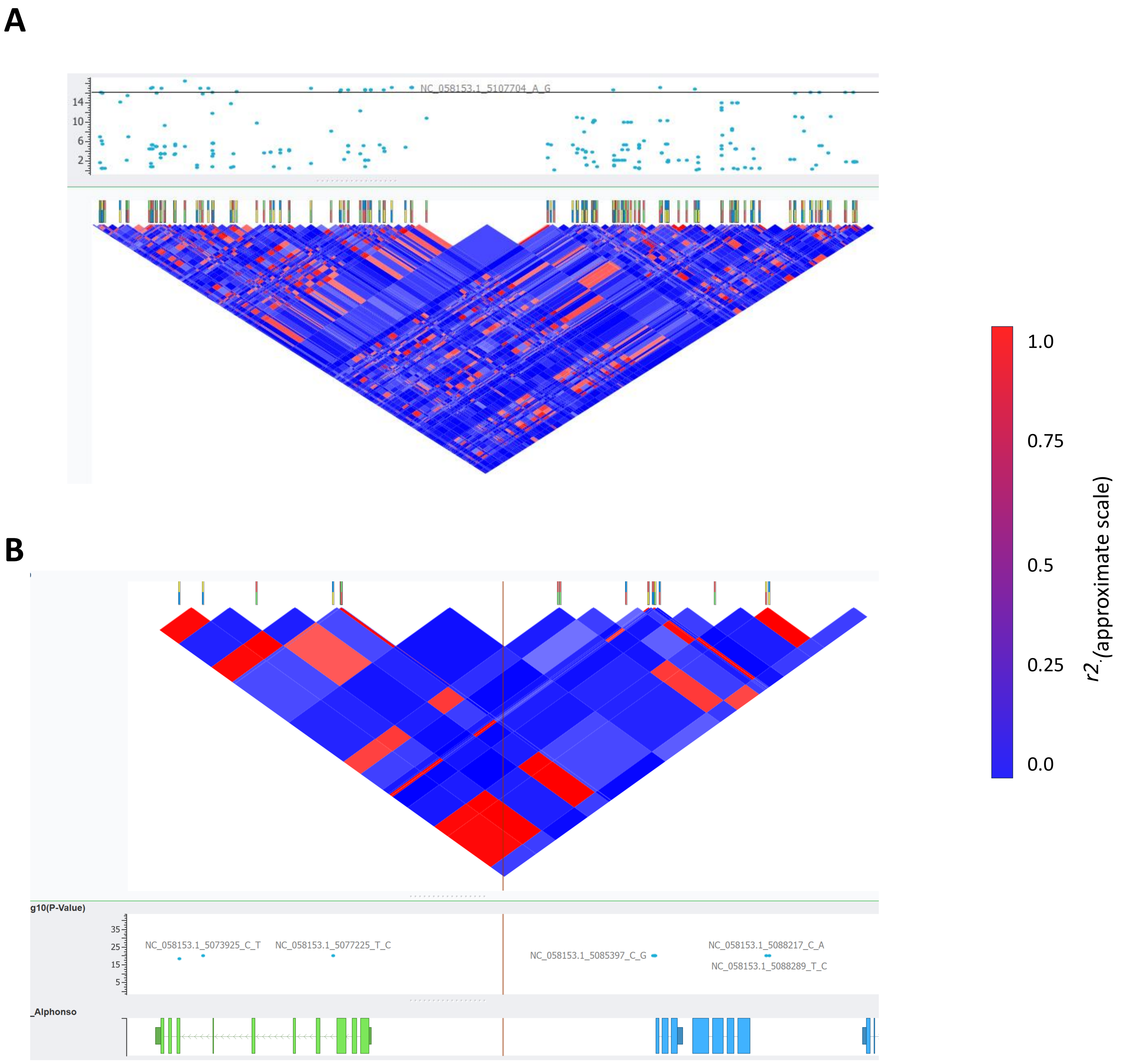

Supplementary Figure S7: Linkage disequilibrium (LD) heat maps of *Mangifera indica* genomic SNPs generated using Golden Helix SVS, illustrating patterns of genetic association and LD blocks.

(A) LD heat map for *M. indica* chromosome 17. The plot shows SNP markers arranged along the chromosome, with linkage disequilibrium visualized as a triangular heat map. Red regions indicate high LD between adjacent SNPs, while blue regions represent low LD. This provides a genome-wide overview of SNP clustering and association patterns.

(B) Zoomed-in view of genomic regions on chromosome 17 strongly associated with polyembryony in *M. indica*. The highlighted regions reveal tightly linked SNPs forming distinct LD blocks, underscoring their potential functional relevance. Gene models are displayed below the heat map, with green and blue bars representing annotated genes in the regions of interest.

A

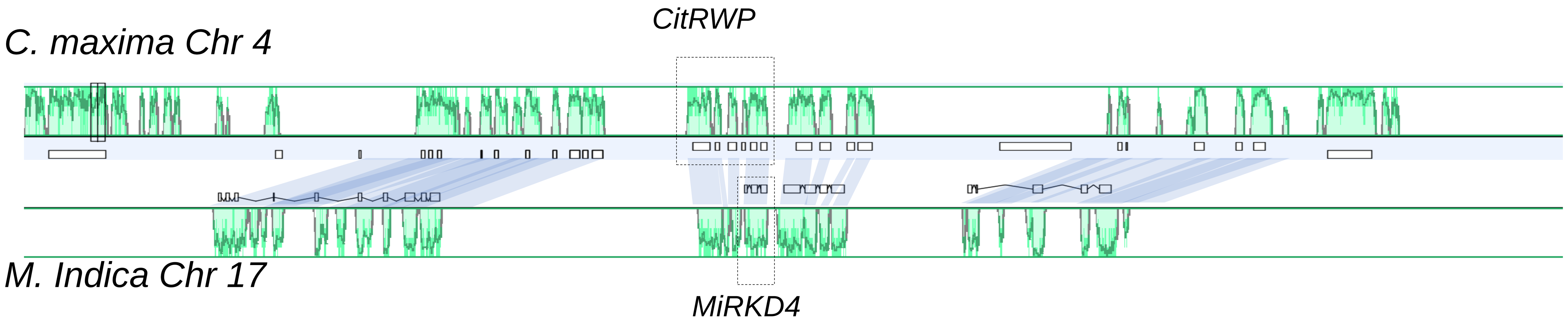

B

```
CitRWP/ CitRKD1  238  MHQPDMENEAERTTRTSLSAQRERTGKLTLRDLMIYFHLPIEEAARRMKLCPTVVKKICR  297
                      M+QP+ E + ER  RTSL+ QRERTGKL L DL  YFHLPIEEAA+R++LCPTVVKKICR
MiRKD4           1    MNQPESEKDGERPPRTSLALQRERTGKLRLGDLRNYFHLPIEEAAKRLRLCPTVVKKICR  60

CitRWP/ CitRKD1  298  RDGLHRWPHRKIKSIQRRMSVASGRLRSNDAEERANAQIEIQRLQEEMAAACAG  351
                      R GL RWPHRKIKS++R++ +A+ R+ S D  ERA A+ EIQRL+ E+  AC G
MiRKD4           61   RYGLLRWPHRKIKSLERQIQIATPRINSIDPAERATAEAEIQRLRHEIDIACTG  114
```

C

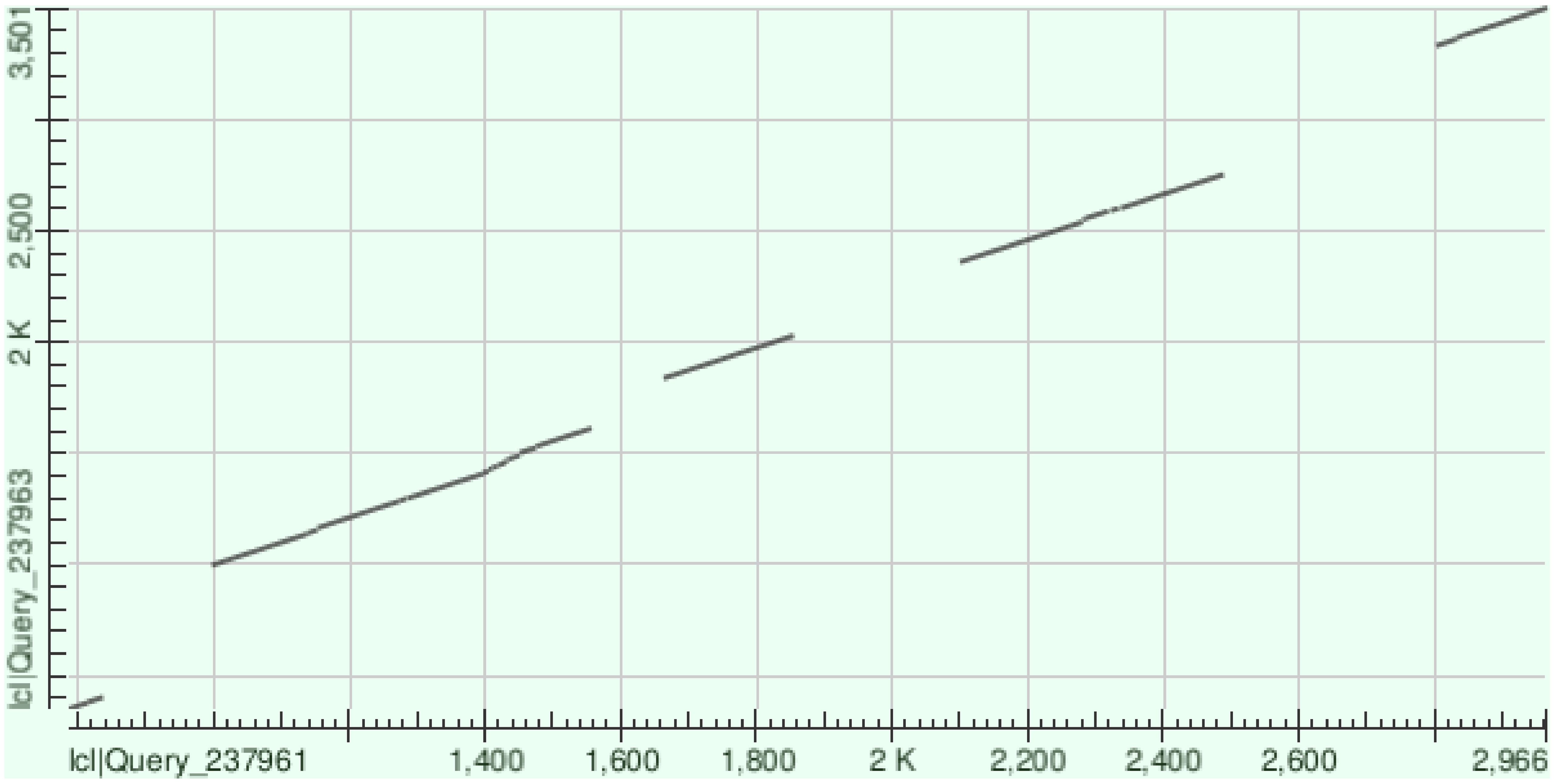

Supplementary Figure S8: Sequence alignment comparison of mango and citrus polyembryonic loci. (A) Synteny plot of polyembryony loci following Mauve alignment: mango chromosome 17 (GCA_011075055.1) vs. pomelo chromosome 4 (GCA_029641205.1). (B) Blastp alignment of mango and citrus polyembryonic genes indicating potential misannotated *MiRKD4* gene with truncated length. (C) Dot plot showing blastn alignment of regions where polyembryonic genes located in mango (GCA_011075055.1) and citrus (GCF_022201045.2). High levels of synteny and collinearity can be seen in this region.

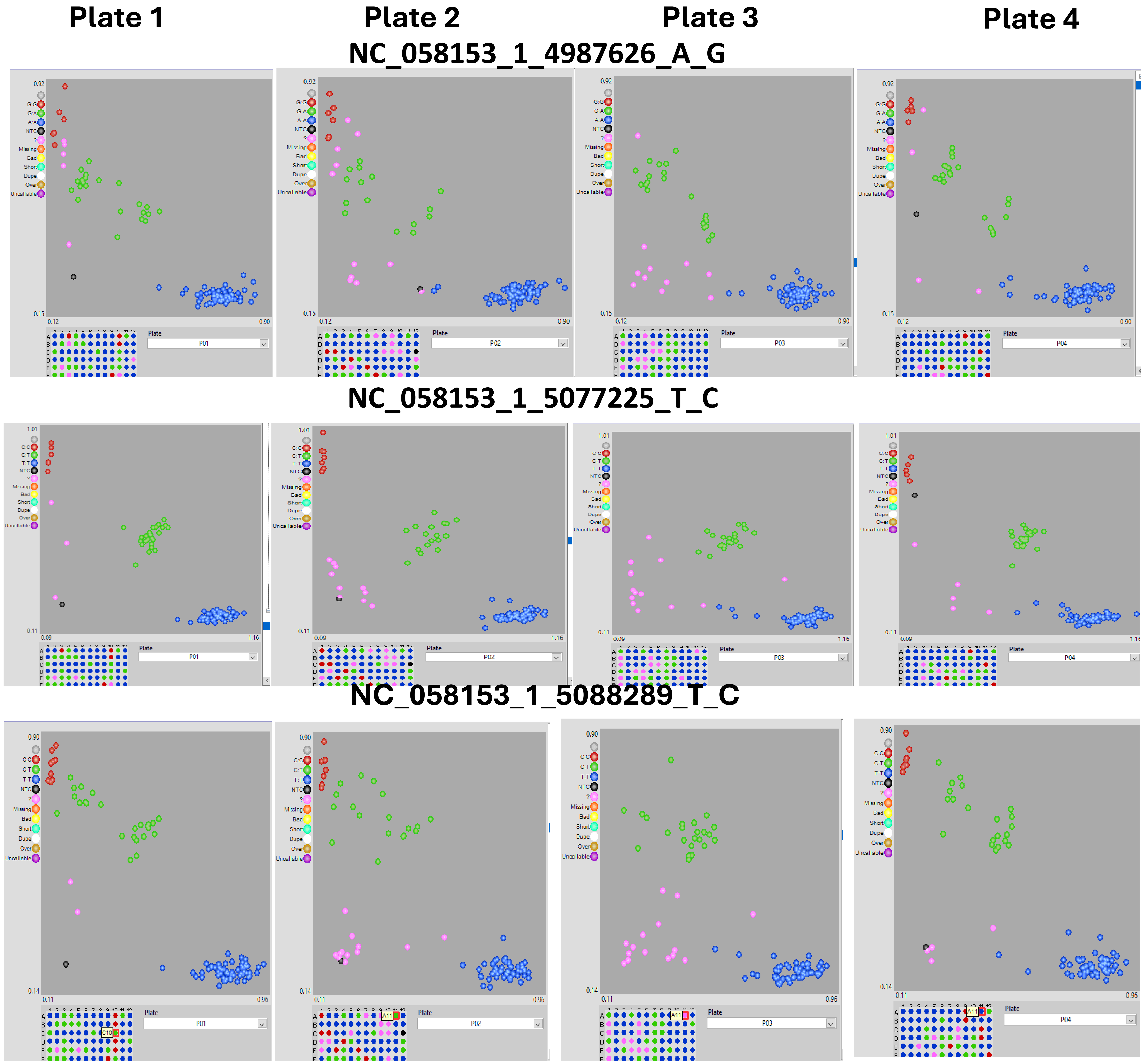
Plate 1
Plate 2
Plate 3
Plate 4
NC_058153_1_4987626_A_G
NC_058153_1_5077225_T_C
NC_058153_1_5088289_T_C
Plate
P01
P02
P03
P04

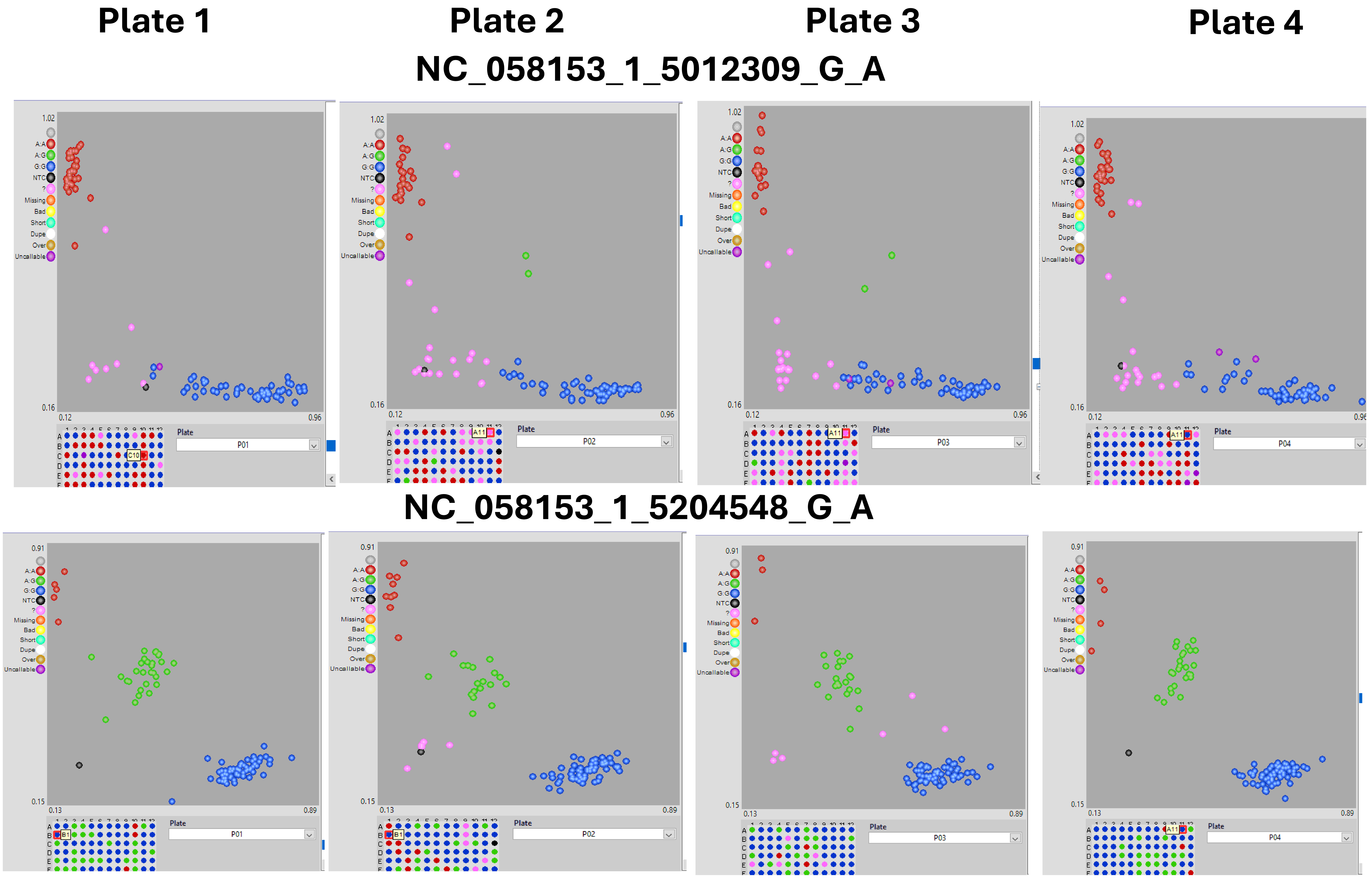

Supplementary Figure S9. The result of KASP genotyping in 380 mango accessions using five KASP markers. The red dots refer to the homozygous polyembryony allele in each marker, the blue dots refer to the homozygous monoembryony allele in each markers , the light blue dots refer to undetermined allele , yellow dots refer to bad call and black dots refer to control samples.

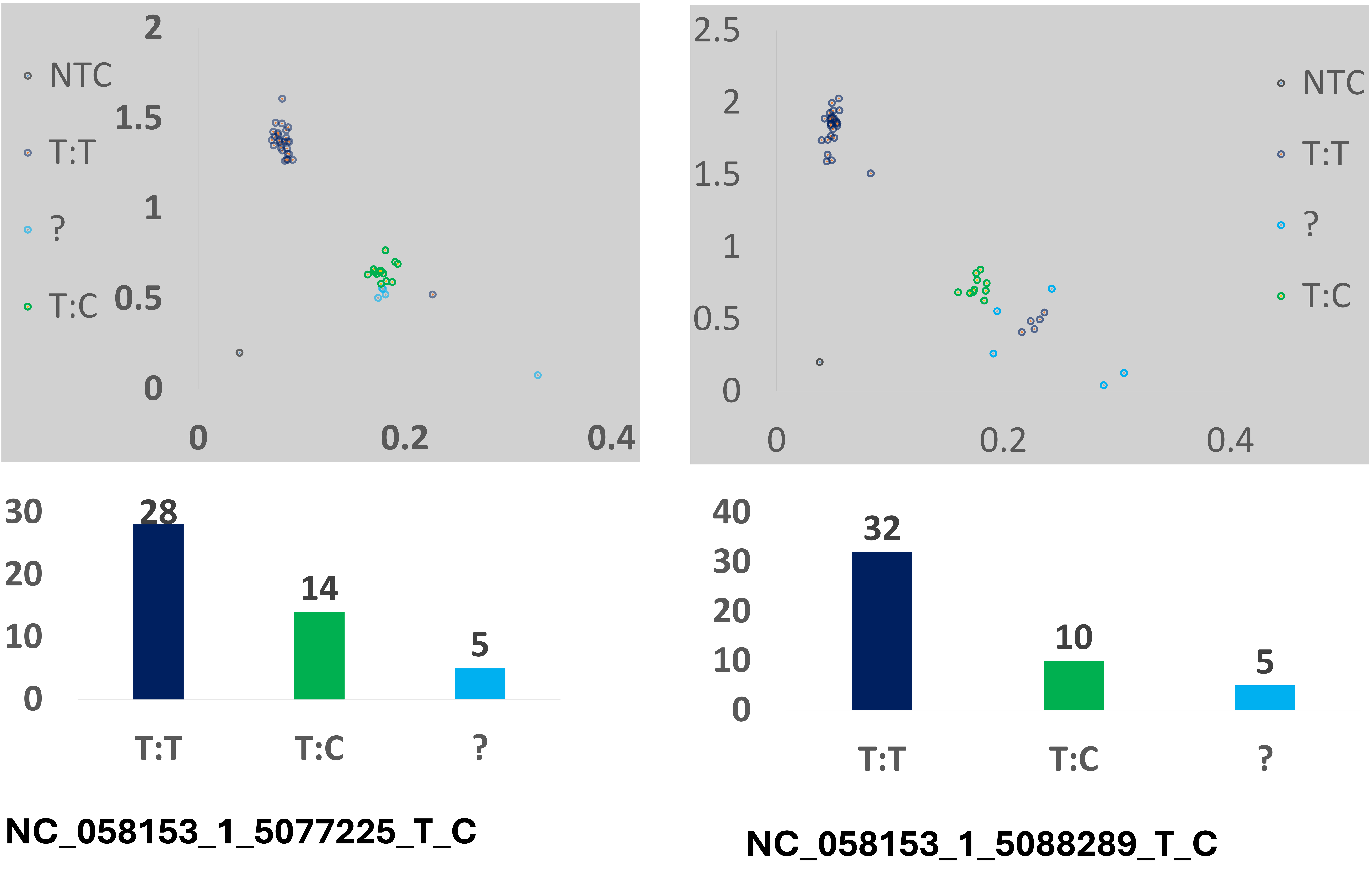

Supplementary Figure S10. The result of PACE genotyping in 47 mango accessions using two KASP markers. the blue dots refer to the homozygous monoembryony allele in each markers and the light blue dots refer to undetermined allele .
